# *In Situ* Synthesis of MXene–Perovskite Interfaces in 3D Carbon Catalysts Boosts Aerobic Oxime Oxidation

**DOI:** 10.1021/acsanm.5c05547

**Published:** 2026-01-21

**Authors:** Elena Romero Salicio, Aicha Anouar, Hermenegildo Garcia, Ana Primo

**Affiliations:** Instituto de Tecnología Química Universitat Politècnica de València-Consejo Superior de Investigaciones Científicas, 16774 Universitat Politècnica de València, Av. De los Naranjos s/n, 46022 Valencia, Spain

**Keywords:** heterogeneous catalysis, catalytic aerobic oxidations, MXenes as thermal catalysts, oxime oxidation, 3D MXenes

## Abstract

The development of heterogeneous catalysts for liquid-phase aerobic oxidation is of great interest. Herein, we report the synthesis of 3D porous graphitic carbon spheres incorporating M_*n*+1_C_*n*_-type MXenes (M = Ti, V, Nb), prepared by delaminating MXene nanosheets in chitosan-based aerogels, followed by pyrolysis. In the case of Nb_2_C, a heterojunction with a NaNbO_3_ perovskite forms within the carbon matrix, leading to the highest catalytic performance. This 3D Nb_2_C/NaNbO_3_ structure achieved a 100% yield in the aerobic oxidation of cyclohexanone oxime to cyclohexanone within 6 h, with negligible metal leaching. Structural analysis revealed the partial oxidation of Nb_2_C to NaNbO_3_ during synthesis, leading to a Nb_2_C–NaNbO_3_ heterostructure. Control experiments confirmed that this interface is essential for the high activity, as neither Nb_2_C nor NaNbO_3_ alone on the porous carbon matrix reached a similar performance. Mechanistic studies based on hot filtration tests, quenching experiments, and EPR spectroscopy demonstrated that the reaction involves reactive oxygen species, mainly superoxide and hydroperoxyl radicals, generated and acting on the catalyst surface. This work provides a promising strategy for designing efficient and robust MXene-based catalysts for sustainable oxidation processes.

## Graphical abstract

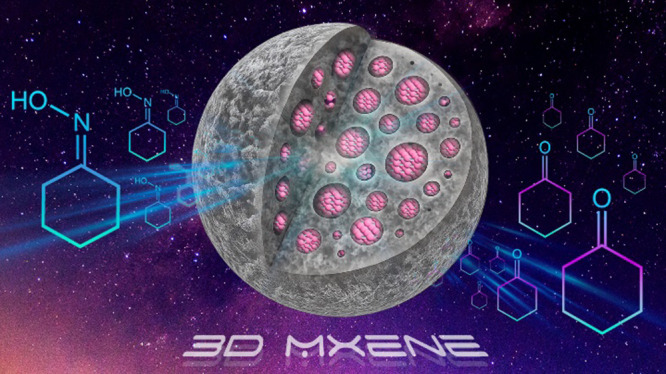

## Introduction

The selective oxidation of oximes to their corresponding carbonyl compounds is an important transformation in organic synthesis, with broad applications in the fine chemical and pharmaceutical industries.^1–4^ In particular, the aerobic oxidation of cyclohexanone oxime to cyclohexanone represents an attractive alternative route for the valorization of oxime derivatives, allowing the generation of valuable cyclic ketones under mild and sustainable conditions.^5^ Traditionally, the catalytic oxidation of oximes has been less explored compared to oxidation reactions consuming stoichiometric amounts of oxidizing reagents. Most reported catalytic oxime oxidation systems rely on noble metal catalysts,^6,7^ strong oxidants,^8^ or harsh reaction conditions, which limit their practical application and raise issues related to environmental compatibility.

In recent years, MXenes, a new family of two-dimensional (2D) transition metal carbides and nitrides (M_*n*+1_X_*n*_T_*x*_), have emerged as promising materials for heterogeneous catalysis due to their unique physicochemical properties.^9–11^ Derived from selective etching of the A-layer in MAX phases,^12^ MXenes combine excellent electrical conductivity,^13,14^ tunable surface chemistry,^15,16^ and a large specific surface area,^17,18^ making them highly suitable as electrocatalysts. Despite their growing use in various catalytic processes,^19–21^ reports on the application of MXenes as aerobic oxidation catalysts are still scarce, and specifically their use for the oxidation of oximes is still unexplored.

One of the main limitations associated with 2D MXenes is their strong tendency to restack or agglomerate due to van der Waals interactions, reducing their accessible surface area and limiting the availability of active sites. To overcome these challenges, the design of three-dimensional (3D) architectures based on MXenes has recently emerged as a promising strategy.^22^ The integration of MXenes into porous 3D carbonaceous frameworks not only prevents restacking but also enhances mass transport and active site accessibility.^23,24^ Moreover, the development of MXene–carbon hybrid materials provides the opportunity to create synergistic effects between the conductive MXene phase and the porous carbonaceous matrix, which improves catalytic performance. In this context, several recent studies have demonstrated the effectiveness of rational 3D integration approaches in enhancing both structure and activity. For instance, Wang et al. developed mesoporous hollow carbon sphere–embedded MXene architectures decorated with Rh nanocrystals, achieving high catalytic performance in electrochemical methanol oxidation due to their large surface area, hierarchical porosity, and excellent electron conductivity.^25^ Similarly, Shen and Huang reported a 3D interweaving Ti_3_C_2_T_*x*_ MXene–graphene network-confined Ni–Fe layered double hydroxide structure with abundant porosity and optimized electronic pathways, resulting in enhanced hydrogen evolution activity.^26^ Furthermore, He et al. constructed 3D interwoven MXene/g-C_3_N_4_/graphene frameworks that exhibit ultrathin porous walls and fast charge transport, effectively maximizing the exposure of active sites.^27^ Thus, although the current available data show how 3D MXene–carbon architectures can serve as versatile and robust platforms for high-performance electrocatalytic systems, examples of an analogous strategy in thermal catalysis are very scarce.

In this context, 3D porous carbon spheres incorporating M_*n*+1_C_*n*_-type MXenes (M = Ti, V, or Nb) were prepared by embedding exfoliated MXene nanosheets into chitosan-derived aerogels, followed by pyrolysis. The catalytic performance of these materials was evaluated in the aerobic oxidation of cyclohexanone oxime to cyclohexanone. The Nb_2_C-based material exhibited the highest activity, achieving complete conversion under mild conditions with negligible metal leaching. Interestingly, detailed structural analysis revealed that during the synthetic process the Nb_2_C MXene undergoes partial oxidation to NaNbO_3_, forming a heterostructure within the carbon matrix that plays a crucial role in enhancing the catalytic performance.

This work provides new insights into the design of advanced MXene-based catalysts, demonstrating that the construction of 3D architectures combining MXenes and their derived oxides within a porous carbonaceous framework offers a versatile approach to the development of efficient and sustainable catalysts for aerobic oxidation reactions.

### Experimental Section

All the information regarding the experimental section, including materials and characterization used in this study, is provided in the Supporting Information.

## Results and Discussion

### Synthetic Procedure

The synthetic approach developed in this work is illustrated in Figure 1 and enables the fabrication of porous carbon spheres embedding M_*n*+1_C_*n*_-type MXenes (M = Ti, V, Nb) homogeneously dispersed within a carbonaceous matrix. The synthesis begins with the selective removal of the A-layer element from the corresponding MAX phase precursors (Ti_3_AlC_2_, V_2_AlC, or Nb_2_AlC) using a fluoride-based etching strategy under different conditions for each precursor. This treatment leads to the formation of multilayered M_*n*+1_C_*n*_ with an accordion-like morphology, characteristic of stacked MXene sheets.

**Figure 1:**
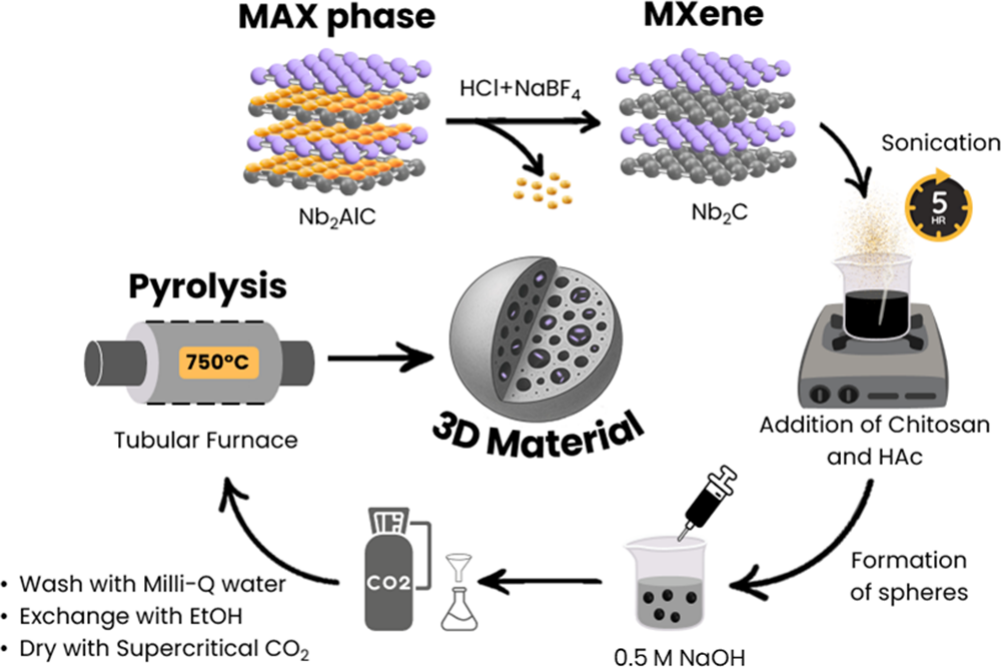
Pictorial illustration of the synthesis procedure of 3D porous carbon spheres embedding M_*n*+1_C_*n*_-type MXenes (M = Ti, V, Nb) homogeneously dispersed within a carbon matrix.

The obtained M_*n*+1_C_*n*_ is subsequently delaminated via ultrasonication in water to produce few-layer MXene nanosheets. These are incorporated into an aqueous chitosan solution under acidic conditions, forming a homogeneous viscous dispersion through favorable electrostatic interactions between the negatively charged MXene surfaces and the protonated chitosan chains.

This dispersion is then introduced into a basic solution to induce gelation, forming chitosan hydrogel spheres with embedded M_*n*+1_C_*n*_ nanosheets. After thorough washing and solvent exchange, the alcogel spheres are dried using supercritical CO_2_ to preserve their porous architecture. Finally, pyrolysis under an inert atmosphere at a moderate temperature transforms the chitosan matrix into a graphitic carbon framework while retaining the well-dispersed MXene phase within the porous spheres.

The resulting materials consist of porous carbon spheres with homogeneously distributed Ti_3_C_2_, V_2_C, or Nb_2_C MXene-derived nanosheets accompanied by their corresponding oxides formed in the process by partial oxidation. These structures exhibit a combination of high specific surface area and well-developed porosity, making them promising candidates for catalytic applications. The embedded MXene phases are stabilized within the carbon matrix, ensuring intimate contact between the metallic component and the carbon framework.

As can be seen in Table 1, the Nb_2_C-derived spheres exhibit a relatively high surface area (260 m^2^ g^–1^), along with a metal content of 7.0 wt %. Figure S1 in the Supporting Information provides the adsorption and desorption isotherms. These isotherms correspond to type-IV profiles with an H3-type hysteresis loop, which is typical for slit-like voids formed by aggregated branches rather than uniform cylindrical pores. The pore size displays a broad distribution from ∼2 to 30 nm, indicating a mesoporous network, consistent with interconnected branches and interparticle voids. Taken together, these features demonstrate a predominantly mesoporous, open structure that explains the high surface area and accessible pore volume. Notably, despite the V_2_C-based material showing the largest surface area, its performance in catalytic tests was significantly hindered by severe leaching of vanadium species under operating conditions, compromising both stability and reusability.

**Table 1:** Physicochemical Properties of the Graphitic Spheres Prepared with Ti_3_C_2_, V_2_C, and Nb_2_C MXenes, Including Their Metal Content (Determined by ICP-OES) and BET Surface Area^a^

material	M (%)	BET surface area (m^2^/g)
3D Ti_3_C_2_	13.8	233.2
3D V_2_C	7.1	320.3
3D Nb_2_C-NaNbO_3_	7.0	260.4

a Summarizes the M content of the 3D MXene samples, while Table S1 in the supporting information lists all the samples prepared in the present study, including those of controls.

Similarly, the Ti_3_C_2_-containing spheres, although structurally robust and moderately porous, showed limited catalytic performance, likely due to extensive surface oxidation of the titanium phase during high-temperature processing and subsequent exposure to ambient conditions. This surface oxidation not only decreases the number of catalytically active sites but may also disrupt the surface electronic interaction between the MXene and the carbon matrix.^28–31^ Surface oxidation of Ti_3_C_2_ should arise from the combined contribution of several factors during sample preparation, including prolonged ultrasound sonication, the use of NaOH in the gelation of chitosan beads, and H_2_O and CO_2_ evolution at a high temperature during the pyrolysis process to convert chitosan into graphitic carbon.

In contrast, the Nb_2_C-based material benefits from a favorable balance of stable metal retention and high surface area. Moreover, Nb_2_C stands out for withstanding hydrolytic and oxidative stress better than Ti- or V-MXenes, a feature that is highly beneficial for enhancing the durability of the Nb_2_C catalyst under harsh reaction conditions.

Catalytic performance of the 3D porous carbon spheres embedding M_*n*+1_C_*n*_-type MXenes was evaluated in the aerobic oxidation of cyclohexanone oxime to cyclohexanone in 6 h at 110 °C (eq 1). Their catalytic activity is summarized in Table 2 which presents conversion and selectivity data achieved for each of the three MXene catalysts. Among the tested materials, the 3D Nb_2_C-NaNbO_3_ catalyst exhibited the highest conversion and selectivity toward cyclohexanone (see Table 2), clearly outperforming its Ti_3_C_2_- and V_2_C-derived analogues.

**Table 2:** Comparison of Conversion, Selectivity, and Yield of the Different Catalysts in the Aerobic Oxidation of Cyclohexanone Oxime to Cyclohexanone^a^

material	conversion (%)	selectivity (%)	yield (%)
3D Ti_3_C_2_ ^b^	43	100	37
3D V_2_C^b^	85	100	78
3D Nb_2_C-NaNbO_3_	100	100	100

a Reaction conditions: cyclohexanone oxime (0.5 mmol), 2 mL of EtOH:H_2_O (1:1), 15 mg of MXene, 110 °C, 6 h. Conversion and yield were determined by GC analysis using dodecane as an external standard.

b Leaching of M (Ti or V) was observed by ICP of the liquid phase.



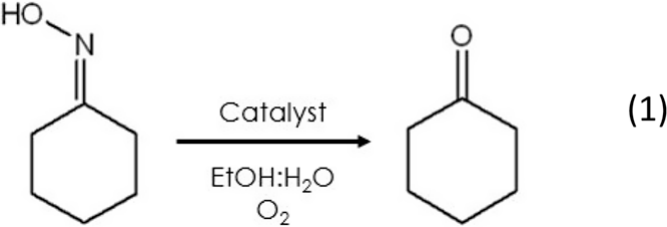

1


This superior catalytic activity can be attributed to the favorable electronic and structural properties of the Nb phases,^32–34^ as well as its homogeneous dispersion within the porous carbon matrix,^35^ which maximizes the accessibility of active sites. In addition, ICP analysis of the post-reaction filtrates revealed negligible leaching of Nb to the solution, confirming the strong support interaction with the Nb_2_C nanophases under the reaction conditions.

In contrast, both Ti- and V-containing catalysts showed significant metal leaching, indicating lower structural robustness and weaker support interaction of the MXene-derived phases with the carbon matrix. This loss of active metal species compromises the catalytic efficiency, recyclability, and long-term application of these catalytic systems. The combination of high activity and negligible leaching underscores the advantage of using Nb_2_C in the design of robust 3D carbonaceous catalysts for liquid-phase oxidation reactions.^36,20^

Given its superior catalytic activity and stability, the 3D Nb_2_C-based material was selected for in-depth structural and morphological characterization. Initial characterization of the carbon-based 3D framework containing embedded Nb_2_C was investigated by X-ray diffraction (XRD). As shown in Figure S2, the diffractogram of the final pyrolyzed spheres does not display any clear reflections attributable to Nb_2_C or any derived phase. This is likely due to the relatively low loading of MXene in the composite (7 wt %), combined with its high dispersion within the amorphous or turbostratic carbon matrix. Additionally, it is not possible to determine from this measurement alone whether the Nb_2_C phase undergoes any structural or chemical transformation during the sequences of gelation, supercritical drying, and pyrolysis.

Fortunately, electron microscopy analysis of the 3D carbon material provided valuable information. Figure 2a shows a HRFESEM image of the multilayered Nb_2_C obtained after selective etching of the MAX phase. The characteristic accordion-like morphology is clearly visible, with increased interlayer spacing resulting from the removal of Al and partial intercalation of termination groups,^37^ consistent with successful formation of the Nb_2_C MXene.

**Figure 2:**
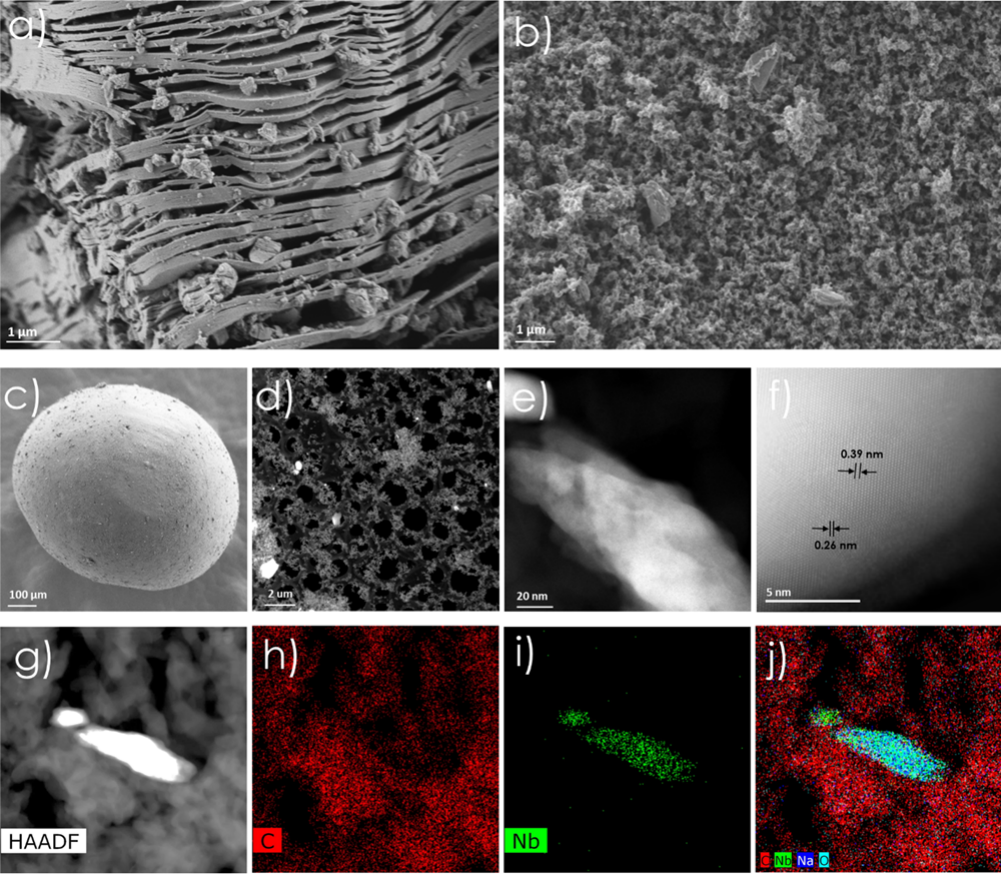
(a) High-resolution FESEM image of multilayered Nb_2_C MXene obtained after Al etching of Nb_2_AlC; (b) FESEM image of the pyrolyzed 3D porous carbon microspheres embedding Nb_2_C–NaNbO_3_ layers; (c) FESEM view of the spherical morphology of a full 3D carbon microsphere; (d) HAADF-STEM image of the Nb_2_C–NaNbO_3_ composite; (e) HAADF-STEM image of the sample showing nanoscale morphology with a 20 nm scale bar; (f) HAADF-STEM image displaying both Nb_2_C and NaNbO_3_ lattice spacings; (g) HAADF-STEM image of the same region used for elemental mapping (scale bar = 80 nm); (h–j) corresponding EDS maps of (h) C, (i) Nb, and (j) composite overlay of C (red), Nb (green), Na (blue), and O (cyan), confirming that NaNbO_3_ flakes are embedded within the continuous carbonaceous matrix.

Figure 2b displays an HRFESEM image of the final pyrolyzed 3D structure, revealing the formation of a porous, sponge-like carbonaceous network. The image also shows the intimate integration of NaNbO_3_–Nb_2_C nanosheets within the carbonaceous matrix, forming a well-interconnected hybrid architecture. This structure is expected to enhance the mass transport and facilitate access to catalytic sites.

Further insights into the crystalline structure of the embedded MXene phase were obtained by high-angle annular dark-field scanning transmission electron miscroscopy (Figure 2d–f). The images show well-defined lattice fringes corresponding to crystalline Nb_2_C domains (Figure 2f). The measured interplanar spacing of 0.260 nm is in agreement with the (100) planes of Nb_2_C,^24^ confirming the preservation in some regions of the MXene crystallinity after pyrolysis. The formation of NaNbO_3_ was also confirmed by HAADF-STEM (Figure 2e). The interplanar distance of 0.390 nm observed for the nanosheets embedded in the carbonaceous matrix corresponds to the (110) plane of orthorhombic NaNbO_3_.^38^ It is worth mentioning that a similar oxidation of Nb_2_C to sodium niobate has previously been reported after an alkali treatment with a sodium hydroxide solution followed by hydrothermal processing at temperatures ranging from 180 to 200 °C.^38,39^ Given that our synthesis protocol involves the use of a basic NaOH solution to coagulate the chitosan spheres, it is likely that some of this sodium remains associated with the MXene, despite extensive washings until pH neutralization. During pyrolysis at 750 °C, a fraction of sodium-associated MXene may undergo oxidation to form NaNbO_3_. To confirm this hypothesis, Nb_2_C was subjected to the same treatment used for the preparation of the spheres, followed by pyrolysis at 750 °C. The formation of NaNbO_3_ at high temperatures was confirmed by XRD. Figure S2 shows the XRD pattern of the resulting material in which diffraction peaks corresponding to the crystallographic planes of orthorhombic NaNbO_3_ can be identified. XRD was also used to further determine in which step during the preparation procedure indicated in Figure 1 appears the NaNbO_3_ phase. Figure S3 gathers these XRD patterns showing that NaNbO_3_ is not formed in any of the steps shown in Figure 1 such as exfoliation, incorporation on chitosan, gelation with NaOH or low-temperature water removal, until the pyrolysis step. During the pyrolysis, the appearance of NaNbO_3_ was observed only at temperatures above 550 °C.

To assess the spatial distribution of the metal component, EDX elemental mapping was performed on a cross-section of the carbon spheres, as seen in Figure 2g–j. The elemental maps clearly indicate the presence of Nb on the particles dispersed in the carbon matrix, confirming the effective entrapment of Nb_2_C particles during synthesis and that NaNbO_3_ flakes are embedded within the continuous carbon matrix. This uniform distribution is key to achieving consistent catalytic performance and minimizing the risk of metal leaching under reaction conditions.

To have a better view of the real 3D structure of Nb_2_C-NaNbO_3_ particles embedded in the carbon matrix, a series of X-Ray microscopy (XRM) images with submicrometer resolution were taken at increasing penetration depths in the 3D microsphere. This set of images was reconstructed using a tomographic software togenerate a three-dimensional view of the microsphere. Supporting Information provides two video clips with these visualizations in every direction of the material. They show a highly porous and open carbon matrix with well dispersed Nb_2_C-NaNbO_3_ particles distributed at all depths and in all directions with the carbon matrix serving to build and hold a 3D network of suspended Nb_2_C-NaNbO_3_ particles.

To evaluate the degree of exfoliation of the Nb_2_C MXene prior to incorporation into the chitosan matrix, atomic force microscopy (AFM) measurements were carried out on the delaminated material after 5 h of sonication in water. As shown in Figure 3, the nanosheets exhibit lateral dimensions in the submicrometer range and thicknesses ranging from 2 to 3 nm, consistent with few-layer Nb_2_C. These results confirm the effective delamination of the multilayered precursor and the presence of well-exfoliated MXene flakes prior to the hybrid material formation.

**Figure 3:**
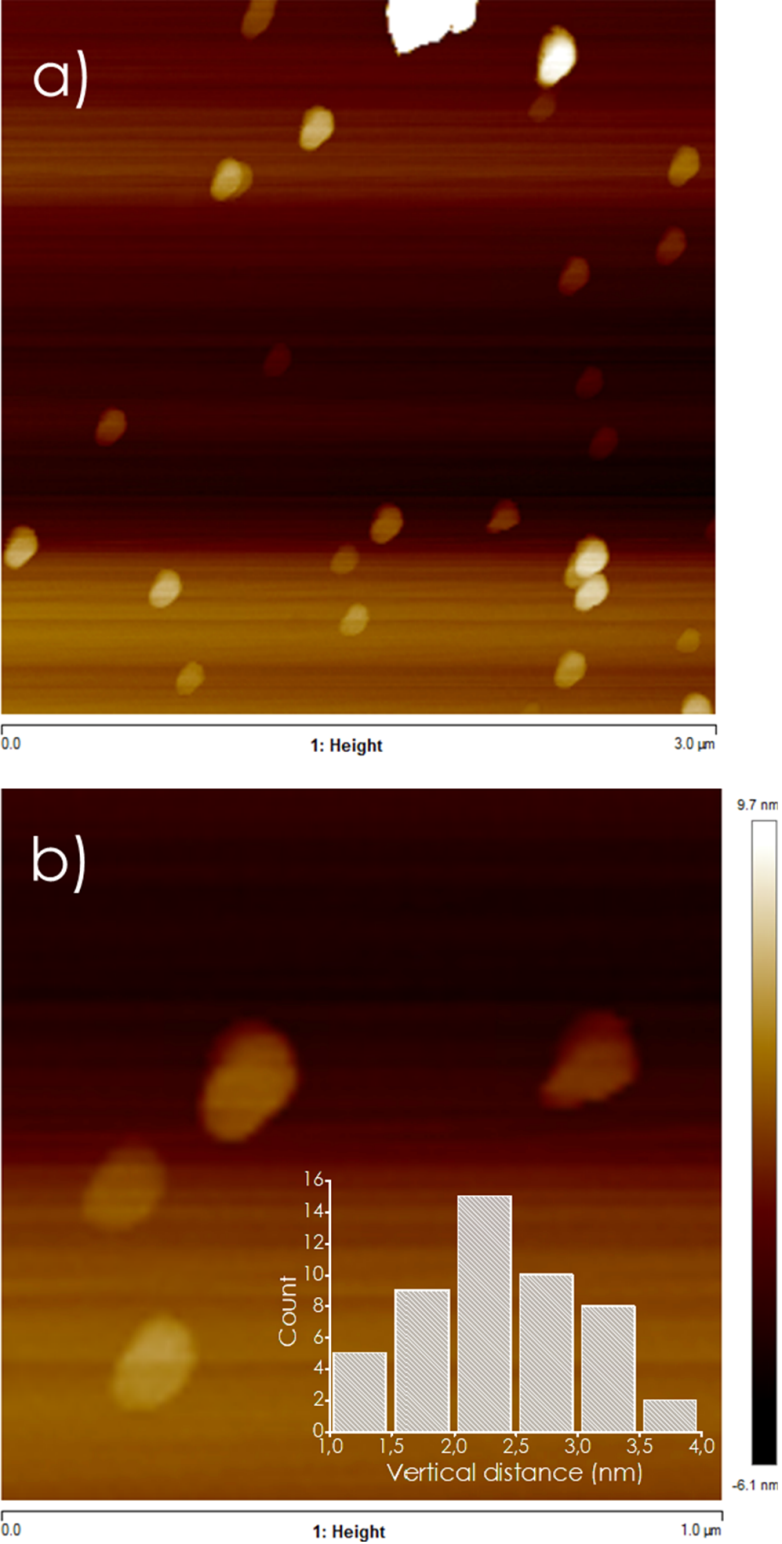
AFM frontal view of Nb_2_C before incorporation into the chitosan matrix: (a) image field of 3.0 (a) and 1.0 μm (b). The inset in frame b corresponds to the histograms of the thickness of a representative number of particles.

However, morphological analysis of the final pyrolyzed spheres suggests that partial restacking or aggregation of the Nb_2_C sheets occurs during the gelation and thermal treatment steps. This phenomenon may result from van der Waals interactions and capillary forces acting during solvent exchange and supercritical drying of the chitosan microsphere as well as from pyrolysis-induced structural rearrangements. Despite this partial reaggregation, the MXene phase remains well-dispersed at the microscale within the carbon matrix, as shown in the tomographic analyses of the material, and retains its crystalline character of Nb_2_C and NaNbO_3_ as confirmed by HAADF-STEM.

X-ray photoelectron spectroscopy confirms that the 3D spheres host a tricomponent heterostructure in which carbide Nb_2_C, perovskite-type NaNbO_3_, and N-doped graphitic carbon coexist in electronic contact, as can be seen in Figure 4. In the C 1s region, the low binding energy signal at ≈283 eV (C–Nb) evidence preserved Nb_2_C layers, while the peak at 284.3 eV (sp^2^ C=C) plus minor C–O/C=O components document a partially oxidized carbon matrix. Nb 3d spectra is not the conventional one for Nb_2_C MXene since the NaNbO_3_ has evolved, and it seems to be preferential on the most external layers of the particles. Thus, the Nb 3d core level displays doublets at 203.7/207.0 (NbC), 204.7/207.7 (NbO_*x*_) and 206.0/207.7 eV (Nb^5+^ in NaNbO_3_), proving partial retention of the carbide MXene and in situ growth of the Nb perovskite. O 1s peaks at 528.3 (NaNbO_3_ lattice), 530.3 (reduced NbO_*x*_/oxy-carbide), and 531.7 eV (C=O/Nb–OH) corroborate this multiphase oxide–carbide interface. Crucially, the N 1s spectrum exhibits a sharp, intense peak at 396.3 eV, characteristic of Nb–N bonds, together with pyridinic (398.7 eV) and graphitic (401.7 eV) nitrogen atoms. The presence of Nb–N indicates partial nitridation at the MXene edges, introducing electron-rich Nb–N sites. The presence of Nb–N, in concert with Nb^5+^/Nb^4+^ centers and conductive Nb_2_C/graphitic domains, indicates the exceptional complex structure of the Nb phases in the material that has to be responsible for its catalytic activity and selectivity in aerobic oxime oxidation.

**Figure 4:**
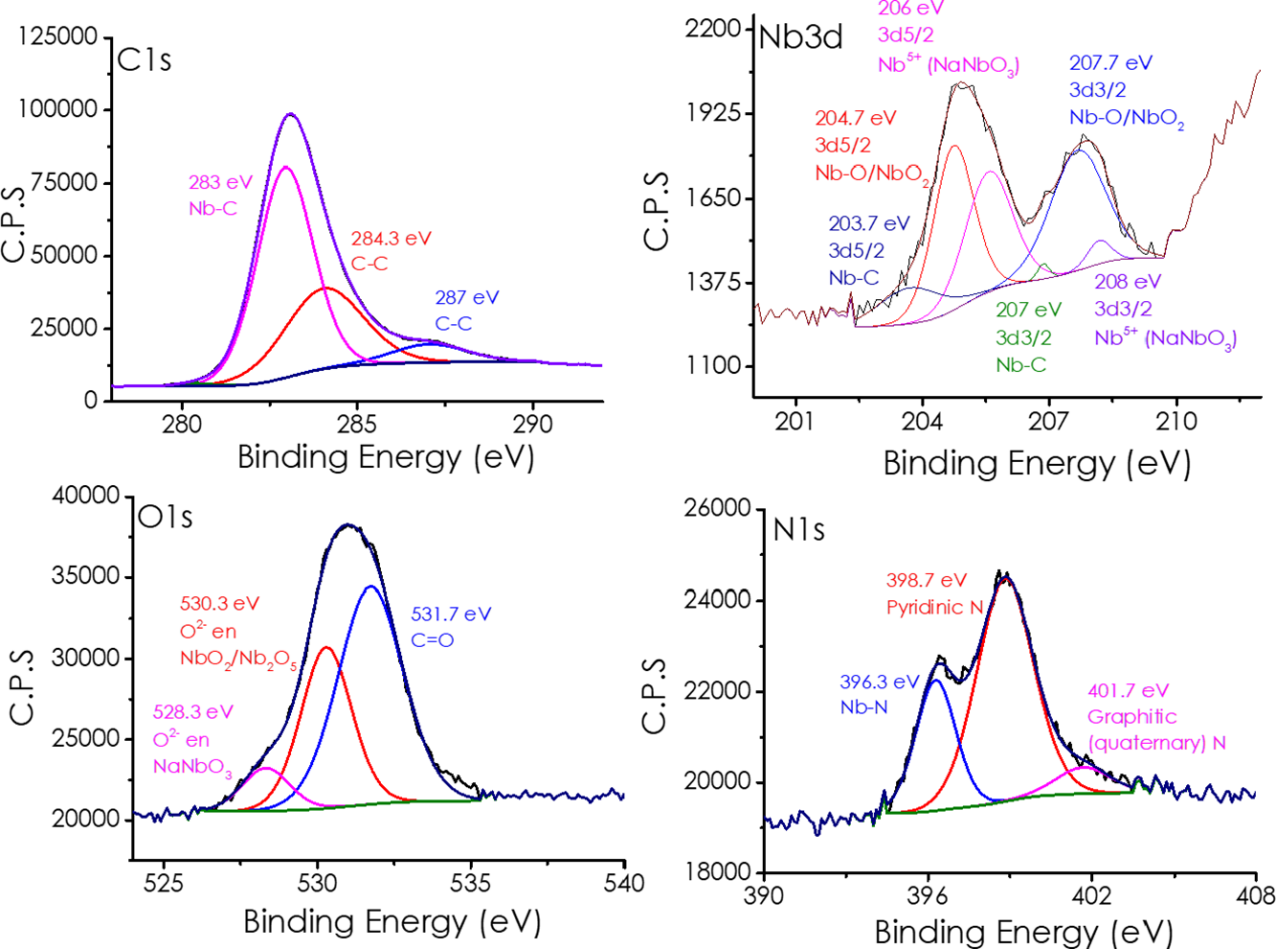
XPS analysis of Nb_2_C-NaNbO_3_ embedded in a N-doped graphitic carbon matrix.

### Catalytic Results

The formation of NaNbO_3_ from Nb_2_C under basic conditions upon heating has been reported in the literature,^38,39^ and it is suggested that the resulting mixed-phase material, comprising both MXene layers and perovskite-type oxide domains, can exhibit enhanced performance due to interfacial electronic effects and synergistic redox behavior of the Nb_2_C-NaNbO_3_ junction. In our system, the coexistence of Nb_2_C and NaNbO_3_ within the carbonaceous matrix may therefore contribute to the high catalytic activity observed in the oxidation of cyclohexanone oxime, as discussed below.

The catalytic activity of the 3D Nb_2_C-NaNbO_3_ based carbon spheres was evaluated in the aerobic oxidation of cyclohexanone oxime in a sealed reactor using a 1:1 ethanol/water mixture as the solvent and cyclohexanone oxime as the substrate. The catalyst was introduced directly into the reaction mixture, which was then purged with oxygen and pressurized to 5 bar with O_2_. The reaction was conducted at 110 °C, and aliquots were periodically withdrawn and analyzed by gas chromatography using dodecane as an external standard.

To assess the role of each component in the catalytic system, a series of blank experiments were performed, as shown in Figure 5a. In the absence of O_2_, under a N_2_ atmosphere, cyclohexanone oxime conversion was below 5%, indicating that the contribution of hydrolysis to the process is negligible. When the reaction was carried out using only Nb_2_C MXene, a modest conversion of cyclohexanone oxime to cyclohexanone (11%) was obtained. Nb_2_C subjected to the same treatment as the hybrid material exhibited a slightly higher activity, reaching a 28% conversion, while commercial NaNbO_3_ led to a conversion of 17%.

**Figure 5:**
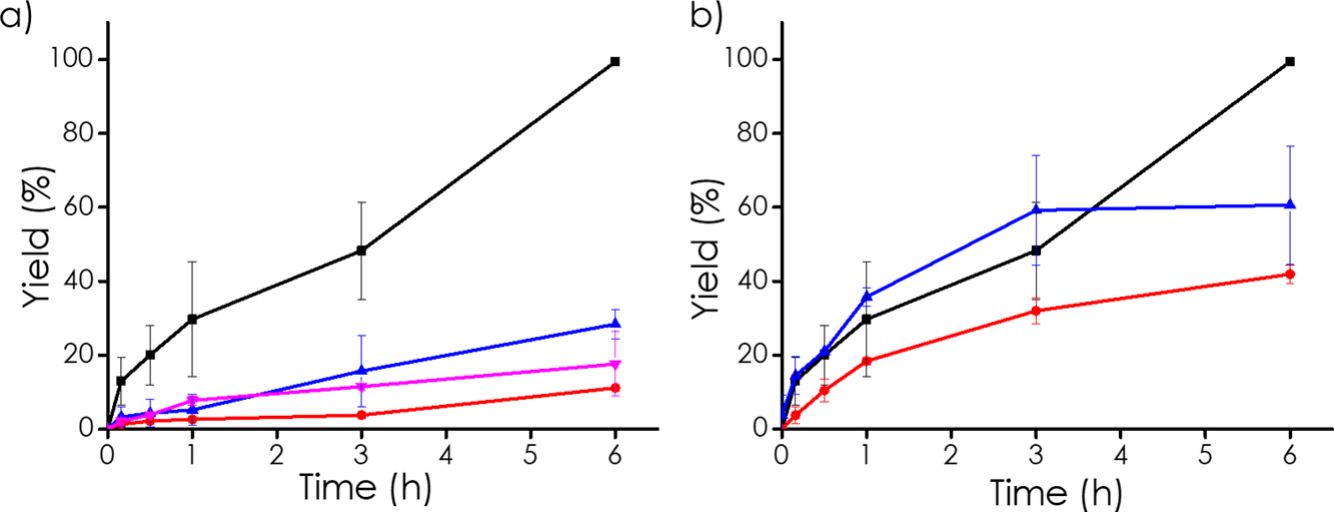
Time–yield plots for the oxidation of cyclohexanone oxime under various conditions: (a) control reactions with different catalysts ■ 3D Nb_2_C-NaNbO_3_ in a carbon matrix, ● Nb_2_C MXene, ▲ Nb_2_C after synthesis treatment, ▼ Commercial NaNbO_3_, and (b) varying MXene loadings in the catalyst ■ 10% Nb_2_C, ● 15% Nb_2_C, ▲ 5% Nb_2_C.

In contrast, the hybrid 3D Nb_2_C-NaNbO_3_ catalyst showed remarkable activity, achieving complete conversion of the substrate within 6 h. These results clearly demonstrate the synergistic effect between the active Nb_2_C and NaNbO_3_ phases and the structured carbon support. The porous carbon network not only provides a high surface area and improved dispersion of the active sites but also enhances mass transport and catalyst stability.^40^ This synergy results in significantly higher catalytic efficiency compared with the individual components alone.

Following the promising catalytic activity observed for the 3D Nb_2_C-NaNbO_3_ based spheres, we proceeded to optimize key reaction parameters, including the MXene loading in the carbon matrix, solvent composition, and reaction temperature. These variables play a crucial role in modulating both the efficiency and selectivity of the transformation.

Further increasing the MXene content in the spheres was initially investigated to assess whether a higher loading would beneficially enhance the catalytic activity. As shown in Figure 5b, the catalyst with a theoretical Nb_2_C loading of 15 wt % (8% of Nb as determined by ICP) exhibited the lowest catalytic activity, despite its higher Nb_2_C content compared to the 10 wt % Nb_2_C theoretical content (7% of Nb as determined by ICP) sample, which achieved complete conversion. This suggests that increasing the Nb_2_C content does not necessarily lead to an improved catalytic performance.

This behavior may result from poor dispersion or aggregation of Nb_2_C at higher loadings, which reduces the number of accessible active sites, as can be seen in Figure S4. Excess Nb_2_C can also become concentrated in specific areas of the porous carbon network, where it forms aggregates that limit the extent of exposed Nb_2_C and thereby limiting its surface exposure and catalytic availability. In fact, effective performance depends not only on total Nb_2_C content but also on its distribution and integration within the 3D carbon framework.

ICP analysis of the washing solution confirmed that a significant fraction of the initial Nb_2_C amount used in the synthesis was not incorporated in the final 3D Nb_2_C-NaNbO_3_ catalyst, suggesting limited uptake capacity of the chitosan-derived matrix. At high precursor concentrations, MXene sheets may sediment or fail to interact efficiently with the support, reducing the level of incorporation. These findings emphasize the importance of balancing MXene and chitosan precursor mass ratios to maximize active site utilization in the resulting 3D Nb_2_C-NaNbO_3_ material.

The choice of solvent was also found to be critical (Figure 6a). Among the tested systems, a 1:1 v/v mixture of ethanol and water resulted in the highest yields. This enhanced performance can be attributed to the dual nature of the solvent system; ethanol improves substrate solubility and facilitates diffusion through the hydrophobic carbon framework, while water may stabilize oxygenated reactive intermediates or participate in proton transfer steps. In contrast, reactions conducted in pure ethanol, toluene, or 1,2-dichloroethane (DCE) led to substantially lower conversions, underscoring the need for a protic and polar medium to support the redox process.

**Figure 6:**
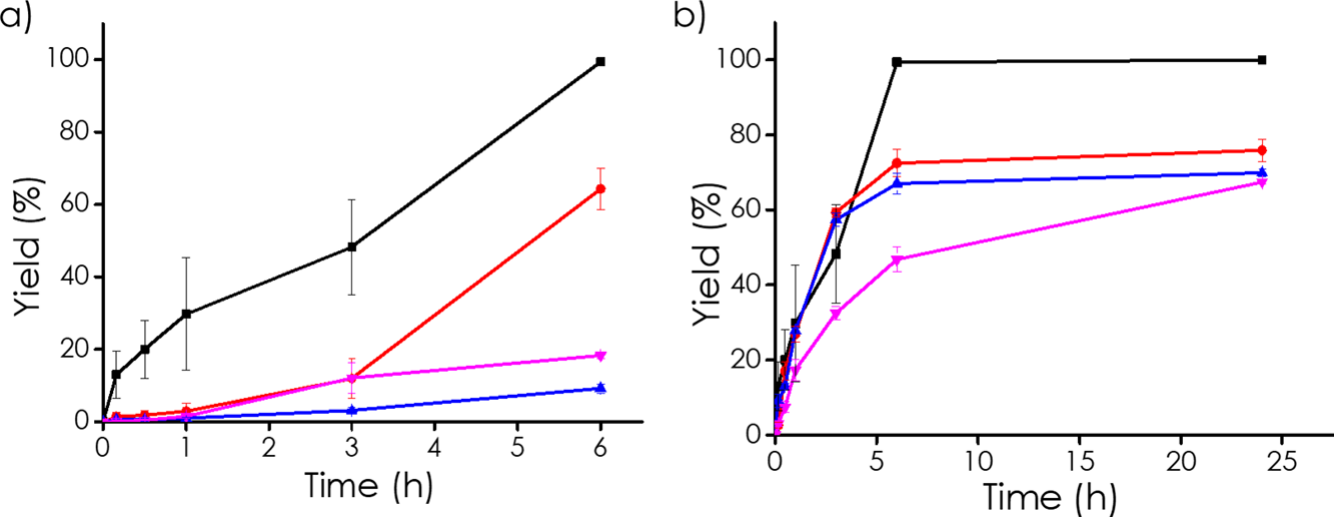
Time–yield plots for the oxidation of cyclohexanone oxime under various conditions: (a) effect of different solvents ■ EtOH:H_2_O (1:1), ● EtOH, ▲ Toluene ▼ DCE, and (b) effect of temperature ■ 110 °C, ● 100 °C, ▲ 90 °C ▼ 80 °C.

Finally, Figure 6b presents the systematic evaluation of the influence of the temperature on the catalytic performance. The reaction rate increased with temperature up to an optimum at 110 °C, beyond which no significant improvement was observed. This temperature was subsequently used for all further studies. From the Arrhenius plot correlating the initial reaction rates with the inverse of the absolute temperature shown in Figure S5, the apparent activation energy (*E*_a_) was calculated. An activation energy of 45.94 kJ/mol consistent with typical values for aerobic oxidation reactions catalyzed by heterogeneous systems was found.

As previously discussed, structural characterization revealed that Nb_2_C MXene undergoes partial oxidation to NaNbO_3_ during the synthesis process, which includes chitosan gelation in NaOH, supercritical CO_2_ drying, and high-temperature pyrolysis. This transformation leads to the formation of a composite material containing both Nb_2_C domains and crystalline NaNbO_3_, which forms a heterojunction within the carbon matrix when the material is pyrolyzed at high temperatures, especially above 550 °C. Such hybrid structures are known to promote enhanced catalytic activity due to interfacial charge transfer and synergistic effects between the metallic and oxide components.^39^

To determine whether the observed high catalytic activity arises specifically from this combination, control experiments were performed using 3D carbon spheres containing only NaNbO_3_ at the same nominal loading as well as 3D spheres prepared solely from carbonized chitosan in order to confirm that the catalytic response was not due to the support alone. In both cases, the catalytic activity was significantly lower, with maximum yields of cyclohexanone reaching only ∼40% after 6 h (Figure 7a).

**Figure 7:**
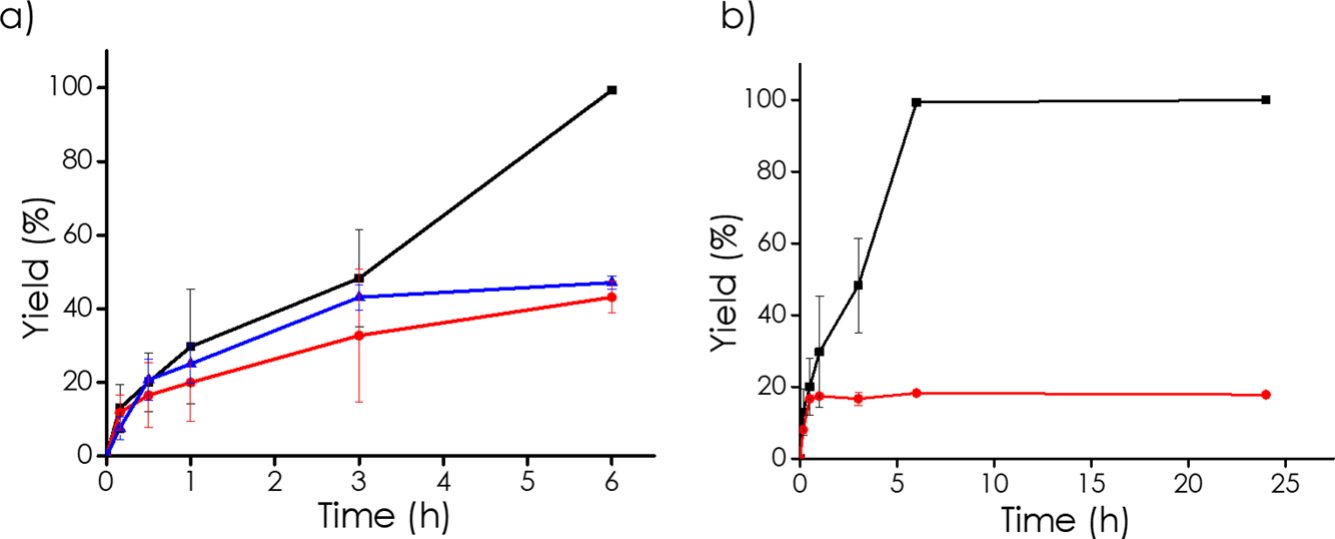
Time–yield plots for the oxidation of cyclohexanone oxime catalyzed by (a) ■ 3D Nb_2_C-NaNbO_3_, ● 3D NaNbO_3_ in a carbon matrix, ▲ 3D carbon matrix; (b) hot filtration test demonstrating that the removal of the catalyst (●) at a given time stops the reaction, in contrast to the uninterrupted progression observed when the catalyst remains in the system (■).

These results confirm that NaNbO_3_ alone cannot account for the exceptional performance observed with the 3D Nb_2_C-NaNbO_3_ catalyst. Rather, the synergy between Nb_2_C and its oxidized counterpart, NaNbO_3_, within the conductive carbon framework appears to be critical. The Nb_2_C–NaNbO_3_ heterojunction likely facilitates improved electron transport and the generation of reactive oxygen species (ROS) under the reaction conditions. Oxygen vacancies on NaNbO_3_ are considered good sites for O_2_ gas adsorption, while the low oxidation state of Nb on Nb_2_C can promote adsorbed O_2_ reduction. Thus, the combination of Nb_2_C and NaNbO_3_ would result in a more efficient oxidative process.

To assess the heterogeneous nature and stability of the 3D Nb_2_C-NaNbO_3_ catalyst under the reaction conditions, a hot filtration test was performed. In this procedure, the oxidation of cyclohexanone oxime was initiated under standard conditions, and after a given reaction time, when partial conversion had been reached, the reaction mixture was rapidly filtered at the reaction temperature to remove the solid catalyst. The resulting filtrate was then returned to the reactor and kept under identical conditions for the remaining reaction time. As shown in Figure 7b, the reaction ceased immediately after the removal of the catalyst, and no further conversion of cyclohexanone oxime was observed. This clearly indicates that the active species remain associated with the solid phase and that no significant leaching of catalytically active components occurs into the solution. These findings confirm the heterogeneous nature of the catalysis and the absence of leaching of active species from the 3D Nb_2_C-NaNbO_3_ solid catalyst to the liquid phase.

To evaluate the stability and robustness of the 3D Nb_2_C-NaNbO_3_ catalyst, recyclability tests were carried out. After each catalytic cycle, the solid catalyst was recovered by filtration, thoroughly washed with ethanol and water, dried, and reused under identical reaction conditions. As shown in Figure S6, the catalyst could be reused for up to four consecutive cycles, observing a gradual decrease in catalytic activity with each reuse.

To investigate the origin of this deactivation, the spent catalyst was characterized by HRTEM, FESEM, XPS, thermoprogrammed oxidation coupled with mass spectrometry detection (TPO-MS), and BET surface area. TEM and FESEM images in Figures S7 and S8 revealed that while the overall porous network of the graphitic spheres was preserved, partial aggregation of the Nb-containing domains occurred after multiple cycles. This aggregation, together with adsorption of organic compounds, would be responsible for the notable decrease of accessible surface area from 260.4 to 71.0 m^2^ g^–1^.

XPS analysis shows the chemical evolution of the 3D Nb_2_C–NaNbO_3_/graphitic carbon upon its use as a catalyst. XPS data of the reused 3D Nb_2_C–NaNbO_3_ catalyst is presented in Figure S9. Relative to the pristine sample, the used material shows in the C 1s region a discernible loss of the 283 eV C–Nb component and a concomitant rise of C=C (284.5 eV) and C=O/C–O signals (288.6 eV), evidencing surface oxidation of both the carbide and the carbon matrix during catalytic cycles. In Nb 3d, the intensity of the 203.7/207 eV Nb–C doublet decreased considerably, whereas the Nb^5+^ doublet at 206.4/209 eV grows, indicating further conversion of Nb_2_C edge sites into Nb_2_O_5_ domains, consistent with the gradual activity loss observed on recycling. Notably alongside pyridinic and graphitic nitrogen, the N 1s spectrum of the used catalyst retains the Nb–N feature but with an upshift from 396.3 to 397.9 eV which is consistent with oxidation of O–Nb–N, confirming that a fraction of electron-rich Nb–N sites survives catalysis and continues to contribute to O_2_ activation.

TPO-MS profiles provide additional important information about the stability of 3D Nb_2_C–NaNbO_3_/graphitic carbon under the reaction conditions. Thus, TPO-MS profiles of the fresh 3D Nb_2_C–NaNbO_3_/graphitic carbon, the pristine graphitic carbon obtained by pyrolysis of chitosan, and the pristine Nb_2_C show that none of these materials undergo oxidation at temperatures below 300 °C. Figure S10 in the Supporting Information provides the corresponding TPO-MS plots as well as the intensity of ions from *m*/*z* 2 to 44 amu. Only a small H_2_O desorption peak (*m*/*z* = 18 amu) at 79 °C was observed for fresh 3D Nb_2_C–NaNbO_3_/graphitic carbon. In comparison, the four times used 3D Nb_2_C–NaNbO_3_/graphitic carbon profile shows a more intense H_2_O desorption peak at 76 °C and a combustion process starting at 180 °C that we attribute to the organic material adsorbed on used 3D Nb_2_C–NaNbO_3_/graphitic carbon due to the contact with reagents. Altogether, the behavior of 3D Nb_2_C–NaNbO_3_/graphitic carbon and its components indicate a notable stability toward oxidation, pointing out that the gradual decrease in activity could be due to a combination of factors, including poisoning of the active sites.

These findings indicate that although the 3D Nb_2_C-NaNbO_3_ catalyst exhibits some reusability, structural and chemical changes at the nanoscale level, such as adsorption of organic compounds, particle agglomeration, decrease in surface area, and surface oxidation, appear to be responsible for the gradual loss of activity observed after repeated use. Given that Nb_2_O_5_ has shown low catalytic activity, we emphasize that the deactivation of the catalyst is likely due to the oxidation of Nb_2_C into Nb_2_O_5_/NaNbO_3_, thereby progressively reducing the catalytic performance of the material with each reuse.

To explore the general applicability of the catalytic system, we investigated the oxidation of a variety of oxime substrates under the optimized reaction conditions. As presented in Table 3, the 3D Nb_2_C-NaNbO_3_ catalyst was tested with structurally diverse aldoximes and ketoximes. Besides aliphatic and alicyclic oximes, the scope also includes aromatic substrates, such as benzaldehyde oxime, *p*-chlorobenzaldehyde oxime, acetophenone oxime, salicylaldoxime, and carvoxime.

**Table 3:** Substrate Scope for the Aerobic Oxidation of Oximes Catalyzed by a 3D Nb_2_C-NaNbO_3_ Catalyst: Conversion of Oxime and Selectivity to the Corresponding Carbonylic Product after 24 h under the Optimized Conditions^a^

reactive	conversion (%)	selectivity (%)	yield (%)
cyclohexanone oxime^b^	100	100	100
benzaldehyde oxime	48	100	45
p-chlorbenzaldehyde oxime	42	100	38
acetophenone oxime	92	100	89
salicylaldoxime	15	100	11
carvoxime	34	100	31
4-methylacetophenone oxime	70	100	66
4-fluoroacetophenone oxime	11	100	10

a Reaction conditions: substrate (0.5 mmol), 2 mL EtOH:H_2_O (1:1), 15 mg of 3D Nb_2_C-NaNbO_3_ catalyst, 110 °C, 24 h. Conversion and yield were determined by GC analysis using dodecane as an external standard.

b Yield obtained after 6 h.

Among all tested substrates, cyclohexanone oxime remained the most reactive, consistently yielding full conversion to cyclohexanone within 6 h. Acetophenone oxime also showed excellent reactivity, affording a yield of approximately 90%, likely due to the stability of the benzylic intermediate and favorable electronic effects. In contrast, benzaldehyde oxime led to lower conversion (∼50%), possibly due to lower nucleophilicity and reduced adsorption on the catalyst surface.

Other aromatic or hindered substrates such as *p*-chlorobenzaldehyde oxime, salicylaldoxime, and carvoxime exhibited even lower reactivities, highlighting the influence of both electronic and steric effects on the oxidation efficiency. Data from the oxidation of substituted acetophenone oximes having a methyl or F group as a substituent in the para position allows one to draw a relationship between the σ_para_ Hammett constant and the initial reaction rate. As shown in Figure S11, no apparent linear relationship between the σ_para_ constant and the reaction rate is obtained, indicating that other factors besides the charge density on the oxime group C atom playing a role in the control of the reaction rate using the 3D Nb_2_C-NaNbO_3_ catalyst. These results demonstrate that while the 3D Nb_2_C-NaNbO_3_ catalyst is broadly applicable, its performance is highly substrate-dependent, with optimal activity observed for cyclic and less sterically hindered ketoximes.

To put into a broad context the results shown in Table 3 about the scope and performance of 3D Nb_2_C-NaNbO_3_ as a catalyst for the aerobic oxidation of oximes, Table S2 provides an overview of the results reported in the literature. As can be seen there, several of the reported catalysts use over stoichiometric amounts of oxidizing reagents to carry out the oxidation, particularly H_2_O_2_ but even adsorbed chromic acid that generates toxic metal wastes in the process. Thus, the present process using O_2_ as the reagent is considerably more advantageous. Regarding the use of O_2_ as oxidizing reagents for the conversion of oximes to carbonylic compounds, most of the studies use 5 bar of O_2_ pressure and frequently temperatures higher than 110 °C, showing again the advantage of 3D Nb_2_C-NaNbO_3_ as the catalyst. One of the most active catalysts is based on Au nanoparticles to activate O_2_ on a defective CeO_2_ support having oxygen vacancies,^41^ while other case is a bimetallic hexacyanocobaltate.^42^ Obvious disadvantages of these two catalysts are the use of precious metal in one precedent and the possible evolution of cyanide in the other one. Based on this analysis of the existing literature, the present data rank 3D Nb_2_C-NaNbO_3_ among the most convenient and best performing catalysts for oxime oxidation to the corresponding carbonyl compound.

### Reaction Mechanism

The fact that O_2_ is required for the reaction to occur indicates that the process is an oxidation rather than an acid–base catalyzed hydrolysis. It should be noted that the density of acid and basic sites in 3D Nb_2_C-NaNbO_3_ is very low and that these sites are weak in strength, as it has been reported for Nb_2_C^20^ and N-doped graphitic carbon.^43^ Aerobic oxidation occurs most commonly through a radical chain mechanism involving ROS. Accordingly, two of the major points in this type of reaction consist of determining the chain length and the nature of the main ROS involved in the reaction. In some cases, there is an initial reaction step in which the first intermediates are formed, and subsequently, propagation steps make that a single event of initiation results in a high number of product molecules due to the long length of the propagation step. Also, frequently these reactions involve free radicals in solution, and the role of the catalyst is just to initiate the chain rather than providing active sites in which the product molecules are formed.

With this background in mind, an initial experiment was carried out in which two twin reactions were carried out under the same conditions, and then at a certain conversion in which sufficient ROS and organic radicals have evolved, the solid catalyst is filtered from the reaction mixture hot in one of the twin reactions. At this point, in the absence of a solid catalyst, the reaction should progress, at least to a certain extent, if the reaction involves free radicals. As shown in Figure 7b, these hot filtration experiments reveal that cyclohexanone formation stops completely upon removal of the solid catalyst. Therefore, this experiment clearly rules out the involvement of free radicals in solution in the formation of cyclohexanone and shows that 3D Nb_2_C-NaNbO_3_ should not be considered as an initiator but is really providing active sites in which catalytic cycles are necessary to form the product.

To learn about the nature of the main ROS responsible for cyclohexanone oxime oxidation, a series of quenching experiments and spin trapping followed by EPR spectroscopy were carried out. Following the literature, a series of twin experiments in the absence of quenchers or adding 10 mol % of a quencher at about 30% conversion were carried out. As quenchers, benzoquinone and DMSO were used as selective inhibitors of superoxide/hydroperoxyl and hydroxyl radicals, respectively. The results of these quenching experiments under the same conditions are presented in Figure 8a. As can be seen there, in the absence of a quencher, the reaction goes to completion at 5 h. In comparison, the addition of benzoquinone at 30% inhibits the reaction that reaches only 55% at 5 h. This means that superoxide and hydroperoxyl are involved as main ROS. In comparison, the addition of DMSO at the same conversion and conditions inhibits the aerobic oxidation to a lesser extent than benzoquinone, reaching a cyclohexanone yield of 78% at 6 h. Based on these experimental data, it is proposed that the interaction of the 3D Nb_2_C-NaNbO_3_ catalyst with molecular oxygen generates superoxide by electron transfer which is the primary ROS species involved in the process. Subsequent protonation by the aqueous medium will form hydroperoxide by a series of electron transfer and protonation will form hydroperoxyl radicals that are also species involved in the oxidation although to about 50% lesser extent. Equations 2–5 summarizes ROS generation and ROS involvement in cyclohexanone oxime oxidation. It has to be commented at this point that comparison of the quenching effect of benzoquinone and DMSO indicates that multiple ROS are present in the reaction mixture during the oxidation, and all of them are able to promote oxidation. The fact that DMSO inhibition is lower than benzoquinone inhibition indicates that superoxide can either evolve toward hydroxyl radicals or directly attack the C=N–OH bond, the two processes occurring approximately in the same extent.

**Figure 8:**
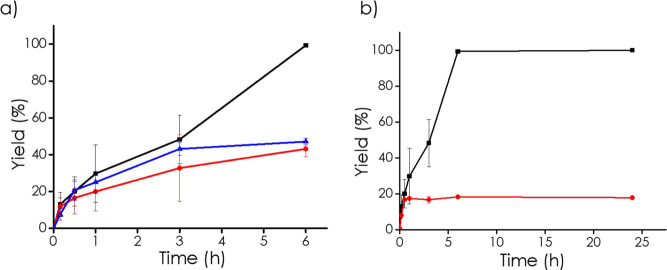
Time–yield plots for the oxidation of cyclohexanone oxime over 3D Nb_2_C-NaNbO_3_ catalyst: (a) oxidation in the ■ absence of quencher, ● presence of Benzoquinone, ▲ presence of DMSO. Note that the quencher was added at 40 min of reaction time. Additionally, the lesser the reaction progresses after 40 min, the more efficient quenching. (b) Catalyst preactivation experiments ■ Standard oxidation under O_2_ atmosphere, ● 15 mg of catalyst preactivated with O_2_, reaction under Ar, ▲ 30 mg of catalyst preactivated with O_2_, reaction under Ar, ▼ 50 mg of catalyst preactivated with O_2_, reaction under Ar at 120 °C.

Spin trapping by 5,5-dimethyl-1-pyrroline N-oxide (DMPO) in water of the ROS generated by the 3D Nb_2_C-NaNbO_3_ catalyst under aerobic conditions was carried out and the experiments were monitored by EPR spectroscopy. While no radicals could be detected for experiments heating the mixture for 5 min, the formation of HOO^·^ radicals was detected after 10 min of heating and characterized by the DMPO-OOH* adduct EPR spectrum (Figure S12). The fine structure of the characteristic EPR spectrum conclusively proves the formation of ·OOH radicals as indicated in eqs 2 and 3. The fact that these ROS species are not detected at shorter heating times is compatible with the occurrence of a certain induction period in the reaction required to generate these ROS on the catalyst surface.



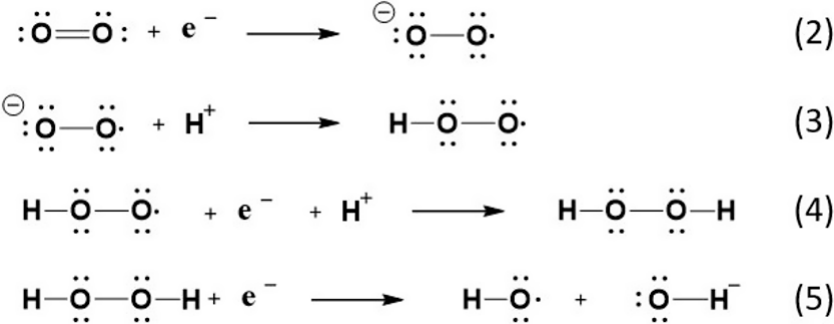

2


After having proved that oxidation requires the presence of a catalyst and that the 3D Nb_2_C-NaNbO_3_ catalyst is not acting as an initiator that only participates in the formation of ROS that subsequently become free radicals in solution, we were interested in confirming that cyclohexanone oxime oxidation occurs on the catalyst surface. To provide experimental support to this hypothesis, 3D Nb_2_C-NaNbO_3_ catalyst was first contacted with oxygen in the absence of cyclohexanone oxime to form the active sites on the catalyst surface, and then, subsequently, this catalyst previously activated by O_2_ was contacted with cyclohexanone oxime under an inert atmosphere under reaction conditions. A blank control in which the same two-step process was performed under identical conditions but the two steps under an argon atmosphere show that no cyclohexanone oxime becomes oxidized to cyclohexanone. In contrast, as shown in Figure 8b, if the 3D Nb_2_C-NaNbO_3_ catalyst is first heated under O_2_, then filtered and added to a cyclohexanone oxime solution under argon, formation of cyclohexanone in 8% yield was observed. This indicates that the number of active sites per gram of 3D Nb_2_C-NaNbO_3_ can be at least 2.7 mmol/g of catalyst.

Considering the available data with the initial generation of superoxide and hydroperoxyl as ROS and the short chain length, it can be proposed that the active sites on the MXene are surface termination vacancies that leave Nb atoms accessible to interact with the atoms of the cationic species in the interaction with O_2_. Formation of a Nb–O_2_ adduct will eventually lead to O_2_^·–^ and concomitant oxidation of the Nb site. Formation of the Nb_2_C-NaNbO_3_ heterostructure will favor this electron transfer to O_2_, because the higher work function of Nb_2_C^44^ will determine electron density migration from NaNbO_3_ with a lower work function^45^ to Nb_2_C. The mechanism will require a subsequent step with the reduction of the Nb atom to the initial oxidation state. This proposal is in line with the current importance of oxygen vacancies on metal oxides for the activation of O_2_ and other species. Spectroscopic studies are necessary to provide some experimental support for this mechanism involving the interaction of molecular O_2_ and Nb with low oxidation states.

## Conclusions

In summary, we have devised a one-pot strategy that converts delaminated Nb_2_C MXene into a three-dimensional N-doped graphitic carbon sphere where the carbide MXene becomes partially oxidized in situ to NaNbO_3_, forming an intimate Nb_2_C-NaNbO_3_ heterojunction. This architecture, stabilized within a high-surface-area, hierarchically porous carbon network, suppresses MXene restacking, maximizes active-site exposure, and endows the catalyst with robustness in liquid media. Among the M_n+1_C_n_ systems investigated (M = Ti, V, Nb), the Nb-based material reaches quantitative conversion and selectivity in the aerobic oxidation of cyclohexanone oxime after 6 h, with no detectable Nb leaching and gradual activity drop after four consecutive uses. Hot-filtration, radical-quenching, and EPR studies confirm that the reaction is strictly heterogeneous and proceeds via superoxide/hydroperoxyl radicals generated at the Nb_2_C-NaNbO_3_ interface. The catalyst also oxidizes a range of aldoximes and ketoximes, reaching 90% yield for acetophenone oxime, highlighting its versatility. These findings establish MXene–perovskite heterostructures embedded in 3D carbon frameworks among the best catalysts for the aerobic oxidation of oximes reported in the literature, and upon further improvement, they can open a new sustainable platform for liquid-phase aerobic oxidation catalysis. To advance further in the use of MXene as a catalyst, future work should develop strategies to increase Nb_2_C stability under the conditions and reagents employed in oxidation reactions.

## Supplementary Material







## Notes

Detailed experimental procedures and materials; synthesis protocols for Nb_2_C, V_2_C, and Ti_3_C_2_ MXenes and their corresponding 3D architectures; additional characterization data including XRD patterns, BET adsorption–desorption isotherms with pore size distributions, HRTEM, and FESEM images, STEM-HAADF micrographs with elemental mapping, XPS and EPR spectra; catalytic reuse studies; and two tables summarizing the physicochemical properties of all materials and reported catalysts for aerobic oxime oxidation (PDF)
X-ray microscopy (XRM) video showing the three-dimensional morphology and spatial distribution of 3D Nb_2_C-NaNbO_3_ (AVI)
X-ray microscopy (XRM) video presenting sequential cross-sectional images through the 3D Nb_2_C-NaNbO_3_ bead, revealing the internal structure and phase distribution of graphitic carbon with Nb_2_C and NaNbO_3_ (AVI)
