# Supplementary material for: In Situ Synthesis of MXene–Perovskite Interfaces in 3D Carbon Catalysts Boosts Aerobic Oxime Oxidation
Source: ACS Appl Nano Mater. 2026 Jan 21;9(4):2074–85. doi: 10.1021/acsanm.5c05547 (PMC12865751; doi:10.1021/acsanm.5c05547)
Supplement: Supplementary file 1 [file an5c05547_si_001.pdf]

Supporting information

In-situ Synthesis of MXene–Perovskite Interfaces in 3D Carbon Catalysts Boost Aerobic Oxime Oxidation

Elena Romero-Salicio[a], Aicha Anouar[a], Hermenegildo García*[a] and Ana Primo*[a]

[a] Instituto de Tecnología Química Universitat Politècnica de València-Consejo Superior de Investigaciones Científicas, Universitat Politècnica de València, Av. De los Naranjos s/n, 46022 Valencia, Spain.

*Corresponding author E-mail addresses: hgarcia@qim.upv.es and aprimoar@itq.upv.es

Experimental

Materials

The MAX phase powders Ti_3AlC_2, V_2AlC, and Nb_2AlC were obtained from NANOCHEMAZONE® with reported purities exceeding 99%. The corresponding batch numbers are as follows: Ti_3AlC_2 – NCZ-07NP/20B, Nb_2AlC – NCZ-01NP/20D, and V_2AlC – NCZ-03MCX/21A.
Hydrochloric acid (37%); lot 2486130 was purchased from Fisher Scientific. Ammonium fluoride; NH_4F (purity ≥99.99% trace metals basis), sodium fluoride (NaF, ACS reagent ≥99%), sodium tetrafluoroborate ($NaBF_4$, purity 98%), Chitosan (Sigma-Aldrich CAS Number: 9012-76-4), Sodium hydroxide (NaOH, ACS reagent ≥98%), acetic acid (CH_3COOH, glacial, ACS reagent, ≥99,7%), Ethanol (≥99.5%, ACS reagent), cyclohexanone oxime (Sigma-Aldrich, purity 97%), cyclohexanone (Sigma-Aldrich, purity 99,8%), benzaldehyde oxime (Sigma-Aldrich, for synthesis), 2-chlorobenzaldehyde oxime (Sigma-Aldrich, purity 98%), Acetophenone oxime (Sigma-Aldrich, purity 95%), Salicylaldoxime (Sigma-Aldrich, ≥98.0%), 5-isopropenyl-2-methylcyclohex-2-enone oxime (carvoxime; Fluorochem), 4'-Fluoroacetophenone oxime, 1-p-Tolyl-ethanone oxime, 4'-Methylacetophenone, 4'-Fluoroacetophenone, (Genochem world S.L.), water-^{18}O (Sigma-Aldrich, 99 atom % ^{18}O) were commercial samples and used as received.

Characterization

Specific surface area of the catalysts was determined by N_2 adsorption isotherms using a Micromeritics 2010 instrument. Field Emission Scanning Electron Microscopy (FESEM) images were acquired using a ZEISS ULTRA 55 microscope equipped with an X-ray detector (EDS). Powder X-ray diffraction (XRD) patterns were recorded on a Cubix Pro PANalytical diffractometer using Cu Kα radiation (λ = 1.5418 Å, 40 kV, 40 mA) in the 2–80° 2θ angle range. HRTEM images were recorded in a JEOL JEM2100F under an accelerating voltage of 200 kV. Specimens were prepared by depositing a microdrop of an aqueous suspension of the material onto a carbon-coated copper TEM grid and allowing it to dry at room temperature. 3D visualization and imaging of the beads were performed using a ZEISS Xradia 620 Versa microscope. Video clips showcasing the 3D

morphology of the as-synthesized beads are provided as supplementary materials. AFM images were recorded using a Bruker multimode microscope under tapping mode. X-ray photoelectron spectroscopy (XPS) data were collected using a SPECS spectrometer equipped with a Phoibos 150 MCD-9 detector employing a nonmonochromatic X-ray source (Al) operating at 200 W. High-angle Annular Dark-Field Scanning Transmission Electron Microscopy (STEM-HAADF) and high-resolution TEM (HRTEM) images were obtained using an FEI Titan G2 (60–300) operated at 300 kV, equipped with a Super-X EDS system.

Synthesis of Nb_2C

Nb_2C MXene was synthesized by selectively etching aluminium from commercial Nb_2AlC MAX phase using procedures previously documented in the literature.[1, 2] Specifically, 1.5 g of sodium tetrafluoroborate ($NaBF_4$) was dissolved in 30 mL of hydrochloric acid (HCl), and 1 g of Nb_2AlC was added slowly during 1 min to the resulting solution inside a 200 mL Teflon-lined stainless-steel autoclave. Adding Nb_2AlC to the HCl and $NaBF_4$ etching solution is an exothermic reaction. Therefore, the MAX phase powder should be introduced slowly and under constant stirring to manage the temperature increase and avoid vigorous reactions. The reaction mixture was maintained at 180 °C for 8 h. After cooling to room temperature, the solid product was recovered by vacuum filtration with a Whatman polyamide membrane filter (pore size of 0.2 µm and a diameter of 47 mm) and thoroughly washed with Milli-Q water until the filtrate reached a neutral pH.

Synthesis of V_2C

V_2C MXene was synthesized by selectively etching aluminium from commercial V_2AlC MAX phase using procedures previously documented in the literature.[3] Specifically, 2.1 g of sodium fluoride (NH_4F) was dissolved in 40 mL of hydrochloric acid (HCl), and 2 g of V_2AlC was added to the resulting solution inside a 200 mL Teflon-lined stainless-steel autoclave. Special care must be taken when adding the MAX phase, as it is an exothermic reaction. Therefore, it is important to add it slowly to prevent a vigorous reaction. The reaction mixture was maintained at 90 °C for 5 days. After cooling to room temperature, the solid product was recovered by vacuum filtration with a Whatman polyamide membrane filter (pore size of 0.2 µm and a diameter of 47 mm) and thoroughly washed with Milli-Q water until the filtrate reached a neutral pH.

Synthesis of Ti_3C_2

Ti_3C_2 MXene was synthesized by selectively etching aluminium from commercial Ti_3AlC_2 MAX phase using procedures previously documented in the literature.[4, 5] Specifically, 5.9 g of ammonium fluoride (NH_4F) was dissolved in 40 mL of hydrochloric acid (HCl), and 1 g of Ti_3AlC_2 was added to a 200 m. Special care must be taken when adding the MAX phase, as it is an exothermic reaction. Therefore, it is important to add it slowly to prevent a vigorous reaction. After complete addition of the Ti_3AlC_2 amount, the mixture was maintained under continuous stirring at 50 ºC for 24 h. After cooling to room temperature, the solid product was recovered by vacuum filtration with a Whatman

polyamide membrane filter (pore size of 0.2 μm and a diameter of 47 mm) and thoroughly washed with Milli-Q water until the filtrate reached a neutral pH.

Synthesis of 3D Nb_2C Structures

To synthesize the 3D Nb_2C, 100 mg of the as-obtained Nb_2C MXene was dispersed in 40 mL of Milli-Q water and sonicated for 5 h. Subsequently, 1 g of chitosan and 1 mL of acetic acid were added to the dispersion, which was stirred for an additional 24 h to ensure complete mixing. The resulting viscous solution was dropwise injected through a syringe and needle into a 0.5 M sodium hydroxide solution to induce gelation and form spherical beads. Once the spheres were formed and stabilized, they were washed with Milli-Q water until the washing water has neutral pH. The wet spheres were then subjected to water-to-ethanol exchange and the resulting alcogel beads dried using supercritical CO_2. Finally, the dried 3D structures were pyrolyzed in a tubular furnace at 750 ºC with a heating rate of 1 ºC/min under inert atmosphere.

Synthesis of 3D V_2C Structures

Exactly the same procedure described for the 3D Nb_2C material was followed to synthesize 3D V_2C, starting in this case with 100 mg of the corresponding exfoliated V_2C MXene.

Synthesis of 3D Ti_3C_2 Structures

The same procedure described for the 3D Nb_2C material was also followed to synthesize 3D Ti_3C_2, starting in this case with 100 mg of the corresponding exfoliated Ti_3C_2 MXene.

Catalytic reactions

A mixture of ethanol and water (1:1 v/v) was used as solvent. To a volume of 2 mL of this mixture, 0.5 mmol of cyclohexanone oxime was initially dissolved. Then, 15 mg of the previously synthesized 3D Nb_2C-$NaNbO_3$ catalyst was added. The reaction system was purged three times with oxygen and subsequently pressurized to 5 bar with O_2. The reaction was carried out at 110 ºC for 6–24 h, depending on the substrate. Reaction aliquots were taken periodically and analysed by gas chromatography. Known amounts of dodecane was used as an external standard. Preliminary reactions were carried out in duplicate, while the rest of reactions were performed in triplicate. Dispersion of the experimental data was lower than 15 %. Data shown in figures correspond to the average and error bars to the standard deviation.

Reuse

Reusability of the same sample of 3D Nb_2C-$NaNbO_3$ was carried out using 0.5 mmol of cyclohexanone oxime, and 2 ml of a mixture of ethanol and water in a 5 ml reactor. Between each catalytic run, the catalyst is recovered by centrifugation and washed three times with ethanol, dried and used in the following run.

Table S1. Textural, structural and synthetic properties of all materials used in this study

Material	Synthetic conditions	Crystalline phase	Morphology	BET Area (m^2g^{-1})	Reference
Nb_2C	Etching 180ºC, 8h	Nb_2C	Nanosheets	5,0	[6]
Nb_2C-NaOH	Nb2C treated in NaOH under 3D synthesis conditions	Nb_2C, $NaNbO_3$, NaOH	Etched flakes	n.d	This work
$NaNbO_3$ (commercial)	Purchased	Orthorombic $NaNbO_3$	Irregular granular particles	n.d	This work
3D Nb_2C-$NaNbO_3$ (Fresh)	Nb_2C + Chitosan pyrolyzed at 750 °C	Nb_2C, $NaNbO_3$	3D porous spherical framework	260.4	This work
3D Nb_2C-$NaNbO_3$ (Reused)	Nb_2C + Chitosan pyrolyzed at 750 °C after reuse	Nb_2C, $NaNbO_3$	3D porous spherical framework	71.0	This work
3D $NaNbO_3$	$NaNbO_3$ + Chitosan pyrolyzed at 750 °C	$NaNbO_3$	3D porous spherical framework	201.2	This work
3D Chitosan	3D Chitosan pyrolyzed at 750 °C	Amorphous	3D porous spherical framework	183.6	This work
3D Ti_3C_2	Ti_3C_2 + Chitosan pyrolyzed at 750 °C	Ti_3C_2, $NaNbO_3$	3D porous spherical framework	233.2	This work
3D V_2C	V_2C + Chitosan pyrolyzed at 750 °C	V_2C, $NaNbO_3$	3D porous spherical framework	320.3	This work

Figure S1. N_2 adsorption-desorption isotherms and pore size distribution of 3D carbon, 3D $NaNbO_3$, 3D $Nb_2C/NaNbO_3$, 3D Ti_3C_2 and 3D V_2C

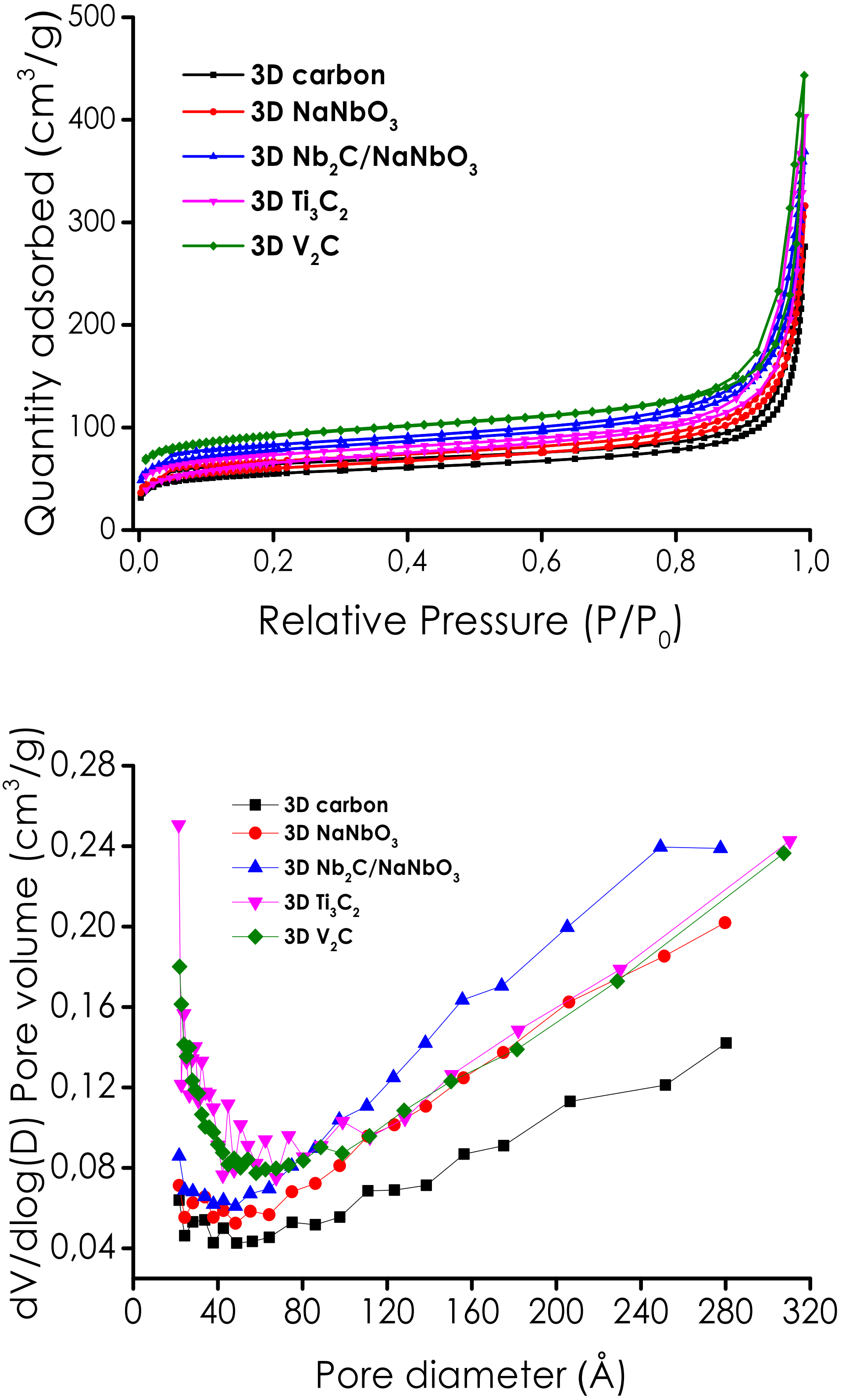

Figure S2. XRD patterns of Nb_2C, Nb_2C-NaOH, Nb_2C-NaOH-750 and 3D $Nb_2C/NaNbO_3$

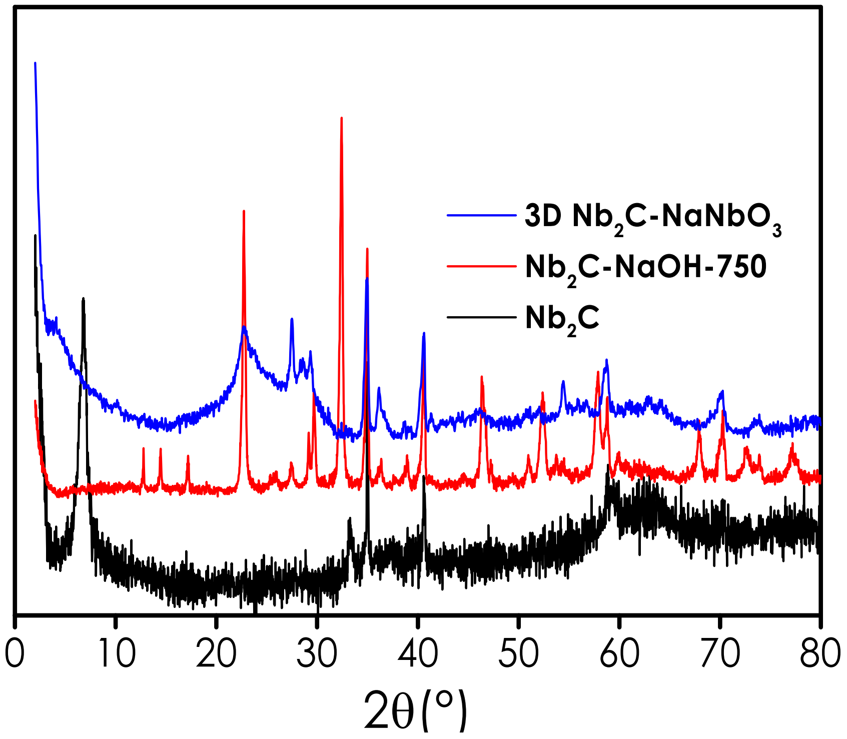

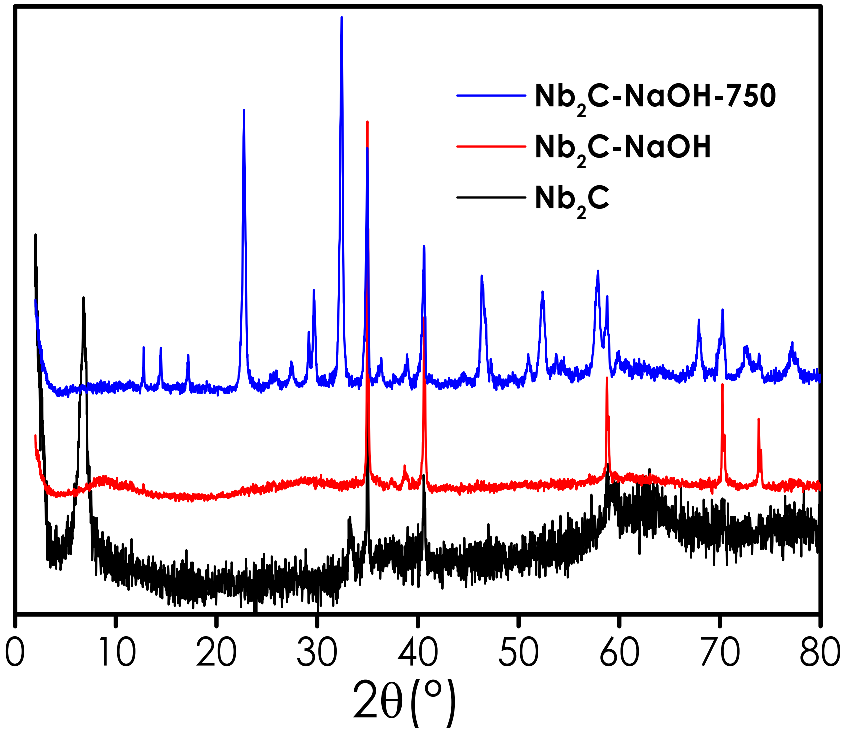

Figure S3. Quasi in-Situ XRD patterns of the catalyst

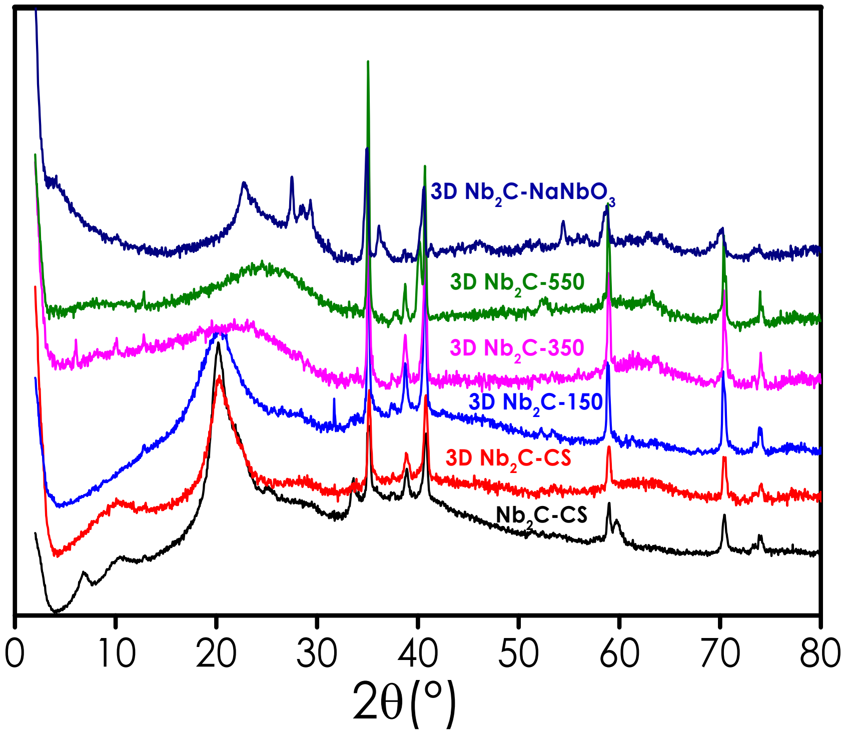

Nb_2C-CS corresponds to the mixture of the chitosan powder and MXene solution.
3D Nb_2C-CS corresponds to the aerogels obtained after supercritical drying.
3D Nb_2C-X corresponds to the material obtained at the temperature X used where X was 150, 350 and 550°C.
3D Nb_2C-$NaNbO_3$ is the material obtained at 750°C.

Figure S4. SEM images of 3D Nb_2C-$NaNbO_3$ based carbon spheres with 15% wt. Nb content that demonstrates the aggregation of Nb respect to the same catalyst with 10 % wt. Nb.

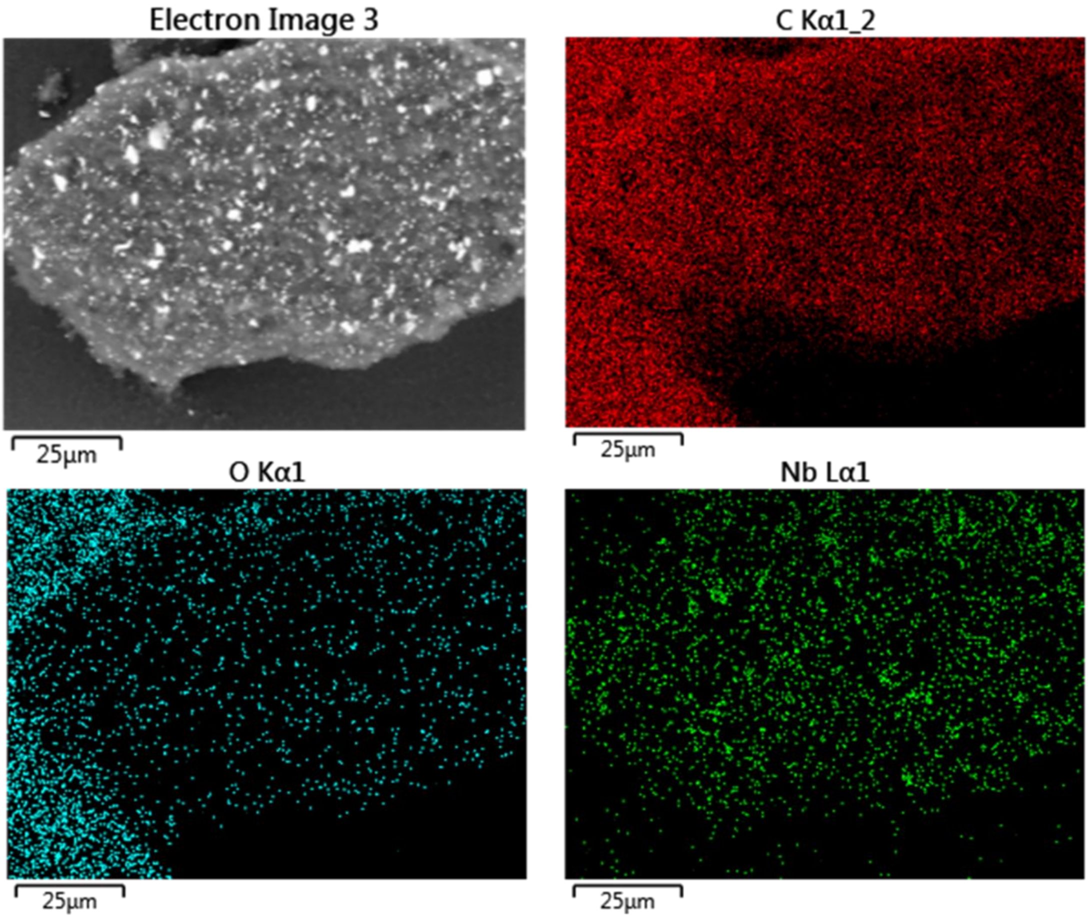

Figure S5. Arrhenius plot correlating the initial rates of the reaction in presence of $Nb_2C/NaNbO_3$ catalyst with the inverse of the absolute temperatures. The activation energy was obtained from the linear fitting of the experimental points. The experimental equation found is y=-5591.8 x + 13.165. Ea=45,94 kJ/mol

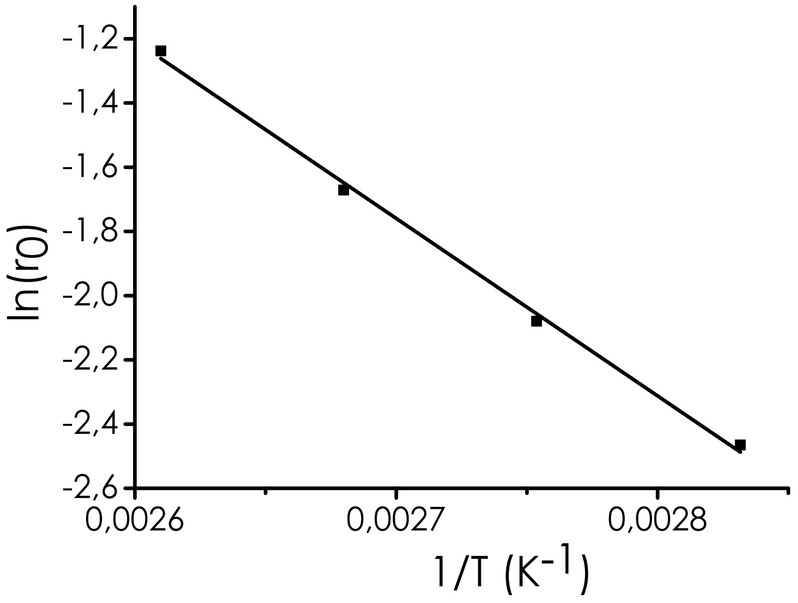

Figure S6. Cyclohexanone yields in a series of consecutive uses of the same sample of 3D Nb_2C-$NaNbO_3$ as catalyst. Reaction conditions: 15 mg of 3D Nb_2C-$NaNbO_3$, cyclohexanone oxime (0.5 mmol), 2 mL of ethanol:water as a solvent, 5 bar O_2, 110°C.

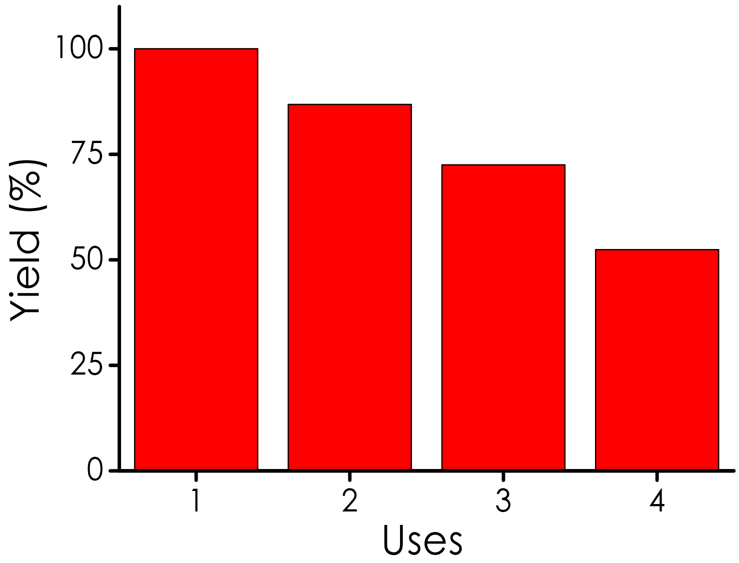

Figure S7. TEM image of a 3D Nb_2C-$NaNbO_3$ sample used four times as cyclohexanone oxime oxidation catalyst. The yellow line corresponds to the interplanar distance of $NaNbO_3$, while the right panel shows the periodic contrast showing the crystallinity of this particle.

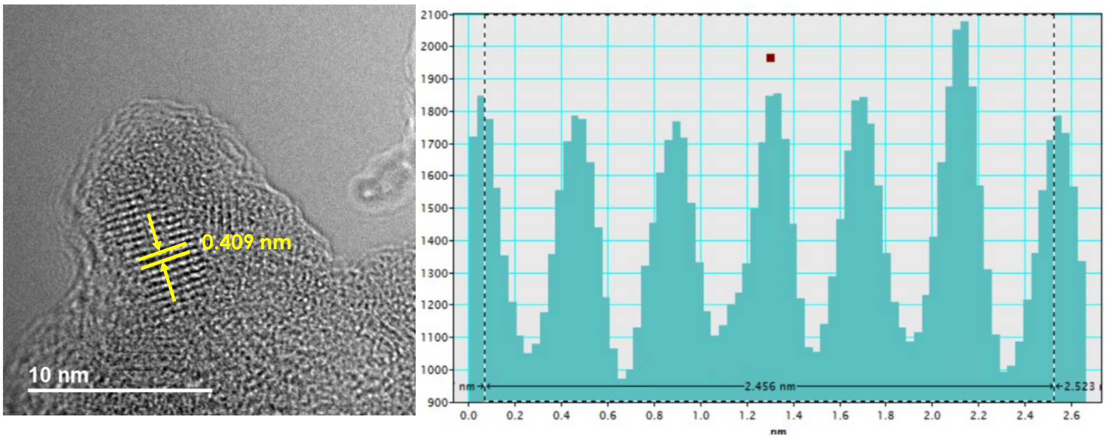

Figure S8. FESEM images of the four times used 3D Nb_2C-$NaNbO_3$ catalyst where it can be observed a partial aggregation of the Nb-containing domains.

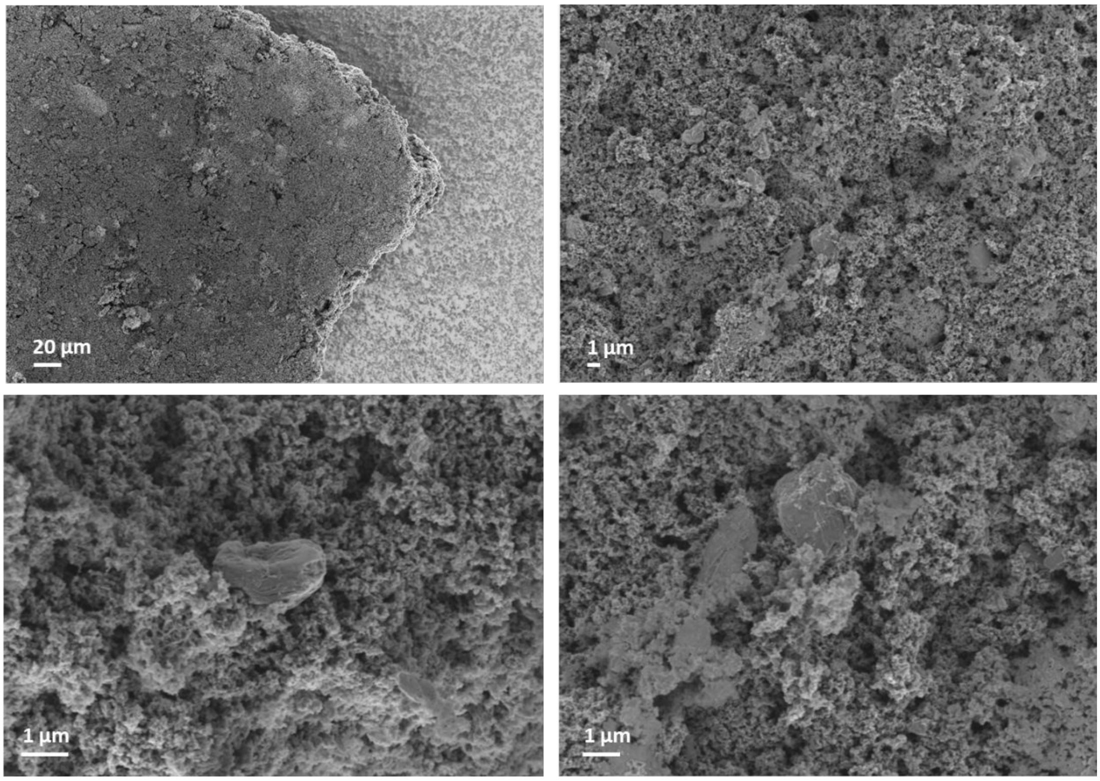

Figure S9. XPS of 3D Nb_2C-$NaNbO_3$ after four consecutive uses as catalyst for cyclohexanone oxime oxidation.

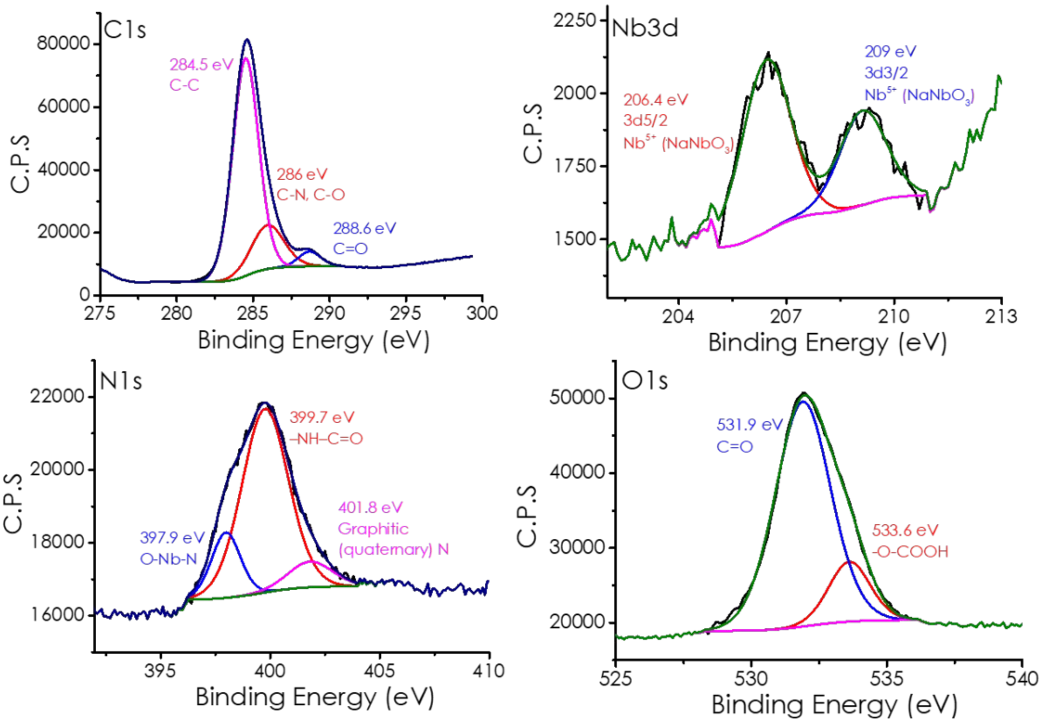

Figure S10. Temperature-programmed oxidation (TPO) profile of the fresh 3D $Nb_2C/NaNbO_3$ catalyst (red) and the catalyst after four uses (blue). The TCD signal shows a single oxidation event with a maximum at ~79 °C for the fresh and at ~76 °C for the used catalyst.

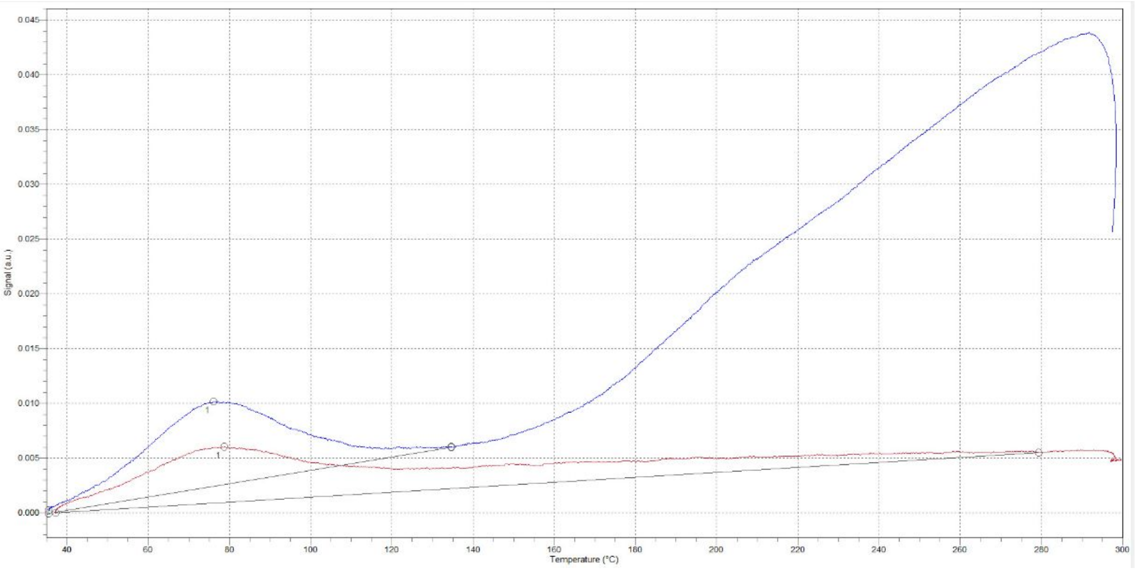

Mass spectrometry signals (m/z = 2–44) recorded simultaneously during the TPO of the **fresh catalyst**. Only minor baseline drifts are observed in the H_2O (m/z 18) and CO_2 (m/z 44) traces at high temperature, with no distinct desorption or combustion peaks. This confirms that the fresh catalyst contains only a small amount of physisorbed water and negligible carbonaceous deposits, consistent with the single low-temperature oxidation feature observed in the TCD profile.

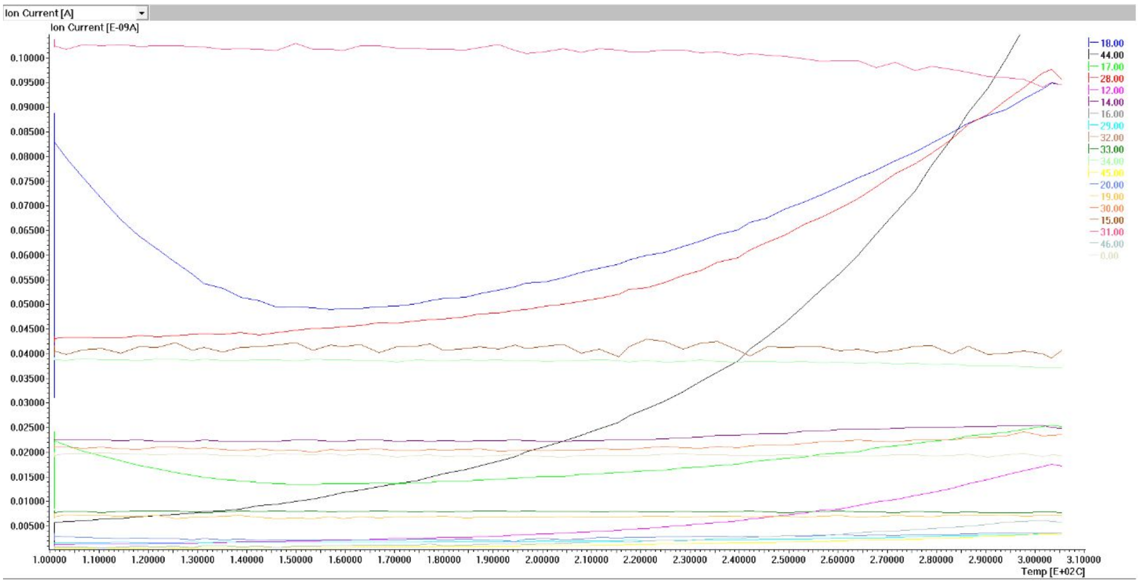

Mass spectrometry signals (m/z = 2–44) recorded during TPO of the **catalyst after four catalytic cycles**. Unlike the fresh sample, the reused material exhibits clear desorption and combustion events, including a distinct H_2O signal (m/z 18) around 200–230 °C and strong increases in CO and CO_2 intensities (m/z 28 and 44) above 220 °C.

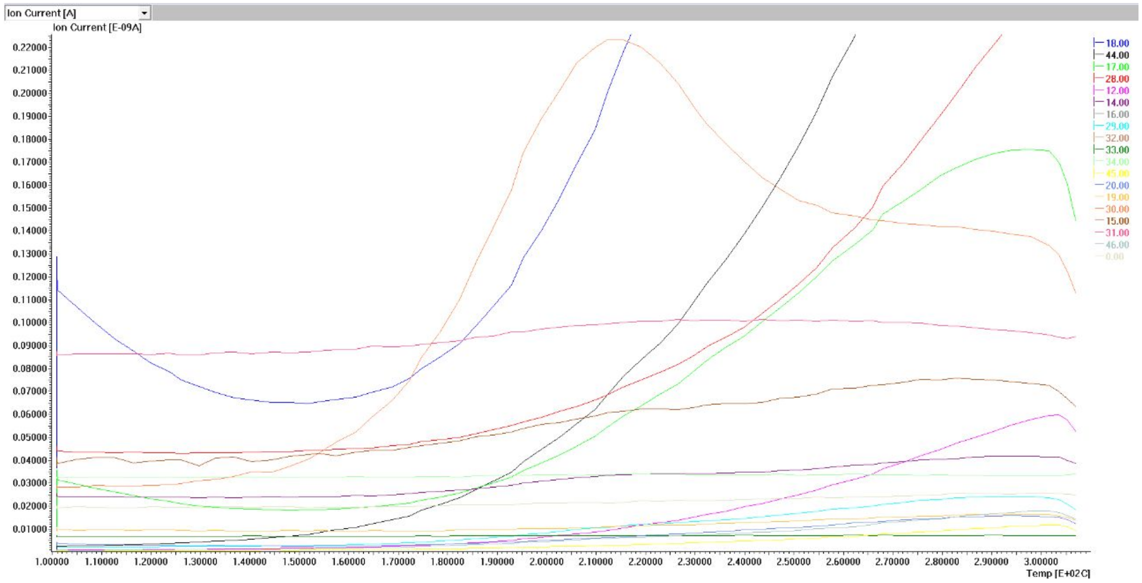
Ion Current [A]
Ion Current [E-09A]
0.22000
0.21000
0.20000
0.19000
0.18000
0.17000
0.16000
0.15000
0.14000
0.13000
0.12000
0.11000
0.10000
0.09000
0.08000
0.07000
0.06000
0.05000
0.04000
0.03000
0.02000
0.01000
1.00000
1.10000
1.20000
1.30000
1.40000
1.50000
1.60000
1.70000
1.80000
1.90000
2.00000
2.10000
2.20000
2.30000
2.40000
2.50000
2.60000
2.70000
2.80000
2.90000
3.00000
Temp [E+02C]
18.00
44.00
17.00
28.00
12.00
14.00
16.00
29.00
32.00
33.00
34.00
45.00
20.00
19.00
30.00
15.00
31.00
46.00
0.00

Figure S11. Hammett plot for the aerobic oxidation of substituted acetophenone oxime using 3D Nb_2C-$NaNbO_3$

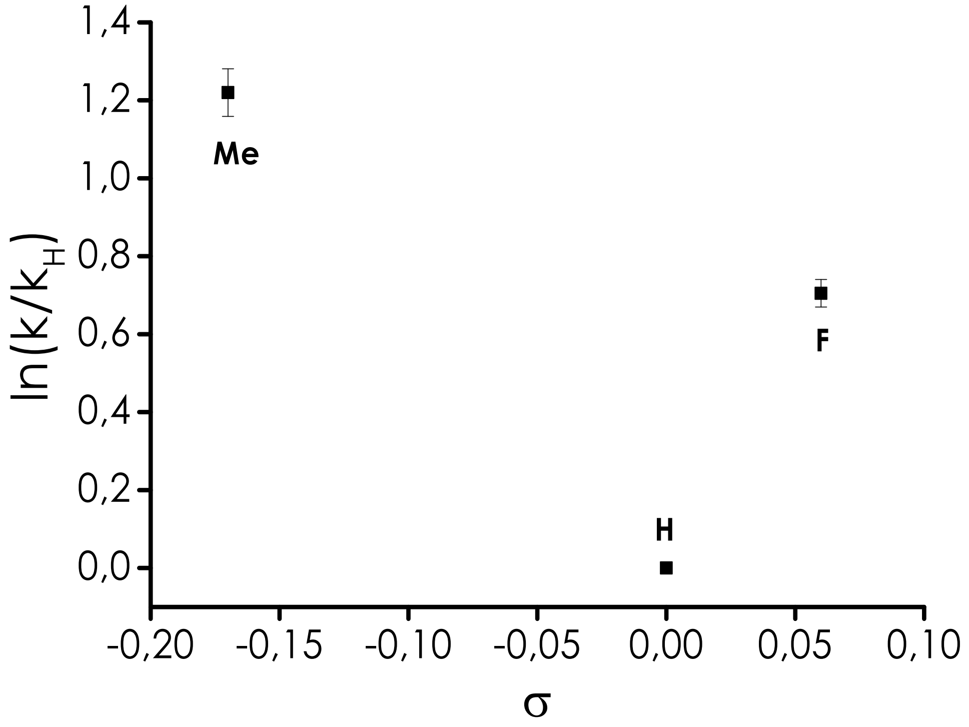

Figure S12. EPR spectra recorded under the following experimental conditions: 10 mg DMPO, O_2 (5 bars), Solvent: EtOH/H_2O (1ml), time 10 min a) blank b) 3D Nb_2C-$NaNbO_3$ catalyst.

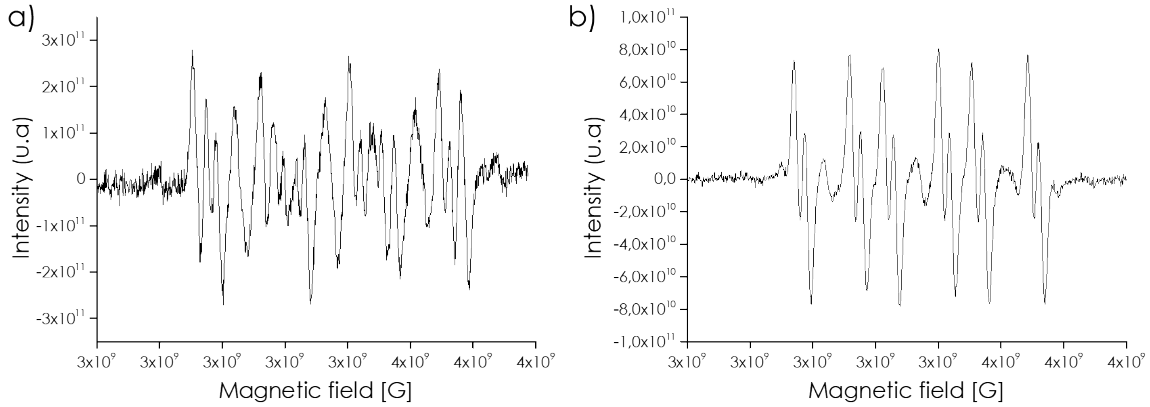

Table S2. Overview of catalysts reported in the literature for oxime oxidation

Oxime substrate	Catalyst	Oxidant	Solvent	Additives	T(ºC)	Conversion (%)	Yield (%)	Time	Reference
Cyclohexanone oxime	$ZrCl_4$/ $PCrO_4H$	Amberlite supported chromic acid denoted as $PCrO_4H$	CH_3CN/H_2O	$ZrCl_4$	Reflux	96	-	55 min	7
Cyclohexanone oxime	PCWP (1mol%)	H_2O_2	$CHCl_3$/ H_2O	-	30	85	93	90 min	8
Benzaldoxime						90	100	80 min	
Acetophenone oxime						80	95	90 min	
Heptylaldoxime						95	70	60 min	
Cyclohexanone oxime	TS-1 (10wt% relative to the oxime substrate)	H_2O_2	Acetone	-	Reflux	-	75	4h	9
Acetophenone oxime							70	4h	
Cyclohexanone oxime	Au (0.72 wt.%)/CeO_x (1mol% Au)	O_2 (5 bar)	EtOH/ H_2O	-	100	99	-	1h	10
Carvoxime					120	99		5h	
Acetophenone oxime					130	99		4h	
Benzaldoxime					130	98		2h	
Cyclohexanone oxime	$Cu_3[Co(CN)_6]_2$ (5mol%)	O_2 (5 bar)	EtOH/ H_2O	-	100	94	82	1h	11
Acetophenone oxime	$FeCu_2[Co(CN)_6]_2$ (5mol%)					100	100	3h	
Cyclohexanone oxime	HKUST-1@CS (8wt% relative to the oxime substrate)	O_2 (5 bar)	EtOH/ H_2O	-	100	-	100	4h	12
Cyclohexanone oxime					130		100	3h	
Acetophenone oxime							89	6h	
Benzaldoxime							92	6h	
Carvoxime					80		82	20h	
Cyclohexanone oxime	Nb_2C-$NaNbO_3$ catalyst (2.25 mol% Nb)	O_2 (5 bar)	EtOH/ H_2O	-	110	100	100	6h	This work
Acetophenone oxime					110	92	89	24h	This work
Carvone oxime					110	34	31	24h	This work
Cyclohexanone oxime	MnTPPCl (1x10^3 mmol)	O_2 (1 atm)	Toluene	Benzaldehyde (15 mmol)	50	93	90	5h	13
Cyclooctanone oxime			Toluene			56	56	8h	
Cyclopentanone oxime			Toluene			85	85	8h	
Acetophenone oxime			Toluene			>99	>99	2h	
Benzophenone oxime			Acetonitrile			98	84	2h	
4-phenyl-2-butanone oxime	$AuBr_3$ (50 mol%)	Au(III) oxidize the hydroxylamine formed in-situ during the reaction	H_2O/EtOH (1:4, v/v)	none	RT	100	-	15h	14
Cyclohexanone oxime	$RuCl_3$ (5mol%)	No external oxidant is required; the oxygen in the carbonyl comes from the oxime	DMA/H_2O = 20:1	PTSA (p-toluenesulfonic acid) – 60 mol%	120	-	86	8h	15
Benzophenone oxime	diphenyl ditelluride $(PhTe)_2$	O_2 (1atm)	Solvent-free	-	Mild (ambient; LED light-driven)	-	93	24h	16
Cyclohexanone oxime	-	$Ce(SO_4)_2$ (3 mmol)	Chloroform	-	RT	-	>90	2h	17
Benzophenone oxime	$(PhCH_2Se)_2$	H_2O_2 (30 wt%), 30 mol% relative to oxime Air (co-oxidant)	Acetonitrile		60	-	65	24h	18

PCWP: $[C_5H_5N^+(CH_2)_{14}CH_3]_3\{PO_4[WO(O_2)_2]_4\}^{3-}$ corresponds to triscetylpyridinium tetrakis(oxodiperoxotungsto) Phosphate
TS-1: Titanium-Silicate
$Cu_3[Co(CN)_6]_2$ and $FeCu_2[Co(CN)_6]_2$ are multimetallic hexacyanocolbatates
HKUST-1@CS is a Cu metal-organic framework embedded into chitosan
MnTPPCl (manganese(III) tetraphenylporphyrin chloride

References

1. Grau, R. R.; Lewandowska-Andralojc, A.; Primo, A.; García, H., Enhancement of the photocatalytic hydrogen production with the exfoliation degree of Nb_2C cocatalyst. *Int. J. Hydrogen Energy.* **2023,** *48*, 20314-20323.
2. Pavel, O.; Tirsoaga, A.; Cojocaru, B.; Popescu, D.; Ramírez-Grau, R.; González-Durán, P.; García-Aznar, P.; Tian, L.; Sastre, G.; Primo, A.; Parvulescu, V.; Garcia, H., Nb_2C Mxene as a bifunctional acid–base and oxidation/hydrogenation catalyst. *EES Catal.* **2025,** *3*, 856-869.
3. Dhakshinamoorthy, A.; Ramírez-Grau, R.; Garcia, H.; Primo, A., Opportunities of MXenes in Heterogeneous Catalysis: V_2C as Aerobic Oxidation Catalyst. *Chem. Eur. J.* **2024,** *30*, e202400576.
4. Ramírez Grau, R.; Garcia-Aznar, P.; Sastre, G.; Goberna-Ferrón, S.; Pavel, O.; Tirsoaga, A.; Cojocaru, B.; Popescu, D. G.; Parvulescu, V. I.; Primo, A.; García, H., MXenes as Heterogeneous Thermal Catalysts: Regioselective Anti-Markovnikov Hydroamination of Terminal Alkynes with 102 h^{-1} Turnover Frequencies. *J. Am. Chem. Soc.* **2025,** *147*, 3315-3332.
5. Anouar, A.; Romero Salicio, E.; García-Aznar, P.; Grirrane, A.; Forneli, A.; Sastre, G.; Garcia, H.; Primo, A., Ti_3C_2 MXene as heterogeneous catalyst for carbodiimides guanylation with over 102 h^{-1} turnover frequencies. *J. Catal.* **2025,** *450*, 116328.
6. Zaheer, A.; Zahra, S. A.; Iqbal, M. Z.; Mahmood, A.; Khan, S. A.; Rizwan, S., Nickel-adsorbed two-dimensional Nb2C MXene for enhanced energy storage applications. *RSC Adv.* **2022,** *12* (8), 4624-4634.
7. Bahrami, K.; Khodaei, M.-M.; Gorgin-Karaji, U., Transformation of Oximes and Alcohols to Carbonyl Compounds Using Amberlite IRA-400 Supported Chromic Acid in the Presence of Zirconium Tetrachloride. *Chin. J. Chem.* **2009,** *27* (2), 384-388.
8. Ballistreri, F. P.; Chiacchio, U.; Rescifina, A.; Tomaselli, G.; Toscano, R. M., Conversion of Oximes to Carbonyl Compounds by Triscetylpyridinium Tetrakis(oxodiperoxotungsto) Phosphate (PCWP)-mediated Oxidation with Hydrogen Peroxide. *Molecules* **2008,** *13* (6), 1230-1237.
9. Joseph, R.; Sudalai, A.; Ravindranathan, T., Selective catalytic oxidative cleavage of oximes to carbonyl compounds with H2O2 over TS-1. *Tetrahedron Lett.* **1994,** *35* (30), 5493-5496.
10. Grirrane, A.; Corma, A.; Garcia, H., Gold nanoparticles supported on ceria promote the selective oxidation of oximes into the corresponding carbonylic compounds. *J. Catal.* **2009,** *268* (2), 350-355.
11. García-Ortiz, A.; Grirrane, A.; Reguera, E.; García, H., Mixed (Fe2+ and Cu2+) double metal hexacyanocobaltates as solid catalyst for the aerobic oxidation of oximes to carbonyl compounds. *J. Catal.* **2014,** *311*, 386-392.
12. Hammi, N.; Chen, S.; Primo, A.; Royer, S.; Garcia, H.; El Kadib, A., Shaping MOF oxime oxidation catalysts as three-dimensional porous aerogels through structure-directing growth inside chitosan microspheres. *Green Chem.* **2022,** *24* (11), 4533-4543.
13. Zhou, X.-T.; Yuan, Q.-L.; Ji, H.-B., Highly efficient aerobic oxidation of oximes to carbonyl compounds catalyzed by metalloporphyrins in the presence of benzaldehyde. *Tetrahedron Letters* **2010,** *51* (4), 613-617.

14. Isart, C.; Bastida, D.; Burés, J.; Vilarrasa, J., Gold(III) Complexes Catalyze Deoximations/Transoximations at Neutral pH. *Angew. Chem. Int. Ed.* **2011,** *50* (14), 3275-3279.
15. Liu, Y.; Yang, N.; Chu, C.; Liu, R., Ruthenium Trichloride Catalyzed Highly Efficient Deoximation of Oximes to the Carbonyl Compounds and Nitriles without Acceptors. *Chin. J. Chem.* **2015,** *33* (9), 1011-1014.
16. Deng, X.; Qian, R.; Zhou, H.; Yu, L., Organotellurium-catalyzed oxidative deoximation reactions using visible-light as the precise driving energy. *Chin. Chem. Lett.* **2021,** *32* (3), 1029-1032.
17. Asutay, O.; Hamarat, N.; Uludag, N.; Coşkun, N., Selective oxidative deoximation with anhydrous Ce(IV) sulfate. *Tetrahedron Lett.* **2015,** *56* (25), 3902-3904.
18. Jing, X.; Yuan, D.; Yu, L., Green and Practical Oxidative Deoximation of Oximes to Ketones or Aldehydes with Hydrogen Peroxide/Air by Organoselenium Catalysis. *Adv. Synth. Catal.* **2017,** *359* (7), 1194-1201.
